# Physiological and transcriptional analyses of developmental stages along sugarcane leaf

**DOI:** 10.1186/s12870-015-0694-z

**Published:** 2015-12-29

**Authors:** Lucia Mattiello, Diego Mauricio Riaño-Pachón, Marina Camara Mattos Martins, Larissa Prado da Cruz, Denis Bassi, Paulo Eduardo Ribeiro Marchiori, Rafael Vasconcelos Ribeiro, Mônica T. Veneziano Labate, Carlos Alberto Labate, Marcelo Menossi

**Affiliations:** Laboratório Nacional de Ciência e Tecnologia do Bioetanol (CTBE), Centro Nacional de Pesquisa em Energia e Materiais (CNPEM), Caixa Postal 6192, 13083-970 Campinas, SP Brazil; Laboratório de Fisiologia de Plantas “Coaracy M. Franco”, Centro de Pesquisa e Desenvolvimento em Ecofisiologia e Biofísica, Instituto Agronômico, Caixa Postal 28, Campinas, 13020-902 SP Brazil; Departamento de Biologia de Plantas, Universidade Estadual de Campinas, Caixa Postal 6109, Campinas, 13083-970 SP Brazil; Laboratório Max Feffer de Genética de Plantas, Departamento de Genética, Universidade de São Paulo, Caixa Postal 83, Piracicaba, 13400-970 SP Brazil; Laboratório de Genoma Funcional, Instituto de Biologia, Universidade Estadual de Campinas Campinas, Caixa Postal 6109, Campinas, 13083-862 SP Brazil

**Keywords:** Sugarcane, Photosynthesis, RNA-seq

## Abstract

**Background:**

Sugarcane is one of the major crops worldwide. It is cultivated in over 100 countries on 22 million ha. The complex genetic architecture and the lack of a complete genomic sequence in sugarcane hamper the adoption of molecular approaches to study its physiology and to develop new varieties. Investments on the development of new sugarcane varieties have been made to maximize sucrose yield, a trait dependent on photosynthetic capacity. However, detailed studies on sugarcane leaves are scarce. In this work, we report the first molecular and physiological characterization of events taking place along a leaf developmental gradient in sugarcane.

**Results:**

Photosynthetic response to CO_2_ indicated divergence in photosynthetic capacity based on PEPcase activity, corroborated by activity quantification (both in vivo and in vitro) and distinct levels of carbon discrimination on different segments along leaf length. Additionally, leaf segments had contrasting amount of chlorophyll, nitrogen and sugars. RNA-Seq data indicated a plethora of biochemical pathways differentially expressed along the leaf. Some transcription factors families were enriched on each segment and their putative functions corroborate with the distinct developmental stages. Several genes with higher expression in the middle segment, the one with the highest photosynthetic rates, were identified and their role in sugarcane productivity is discussed. Interestingly, sugarcane leaf segments had a different transcriptional behavior compared to previously published data from maize.

**Conclusion:**

This is the first report of leaf developmental analysis in sugarcane. Our data on sugarcane is another source of information for further studies aiming to understand and/or improve C_4_ photosynthesis. The segments used in this work were distinct in their physiological status allowing deeper molecular analysis. Although limited in some aspects, the comparison to maize indicates that all data acquired on one C_4_ species cannot always be easily extrapolated to other species. However, our data indicates that some transcriptional factors were segment-specific and the sugarcane leaf undergoes through the process of suberizarion, photosynthesis establishment and senescence.

**Electronic supplementary material:**

The online version of this article (doi:10.1186/s12870-015-0694-z) contains supplementary material, which is available to authorized users.

## Background

Sugarcane is a tropical crop with C_4_ photosynthetic metabolism and Brazil is its main producer. World’s interest in bioethanol and sugarcane with high sucrose and biomass yield has increased continuously over the years. In fact, sugarcane is one of the most important crops used as sustainable feedstock for renewable energy, such as bio-electricity and bioethanol, in tropical and subtropical regions (International Energy Agency - http://www.iea.org). Worldwide, sugarcane is cultivated in 22 million ha and its average yield is 70.9 Tonnes/ha [1]. This crop is very efficient in intercepting solar energy and assimilating carbon into carbohydrates, which results in high sucrose accumulation (around 0.7 M) in its mature culms and high biomass production [2]. However, the current average yield is less than 20 % of the theoretical maximum estimated from mathematical models of plant growth and physiological processes [3]. Photosynthesis plays a key role on biomass production and crop yield, however, our understanding about this important physiological process in sugarcane is more limited when compared to maize, another C_4_ species. This highlights the need for detailed sugarcane physiology studies, particularly on leaf photosynthesis. Such efforts would aid bridging the gap between the average yield in the field and the theoretical maximum yield of sugarcane.

Modern sugarcane originated from inter-specific hybridization between the parental species *Saccharum officinarum* L. (2n = 80) and *S. spontaneum* L. (2n = 40–128). Despite its economic importance, the complexity of the sugarcane genome [4–6] with its haploid genome size estimated of 930 Mbp and high ploidy, aneuploidy and polymorphism, [4, 7, 8] have limited the advances on the development of new varieties via molecular breeding approaches. Classical breeding has been the only responsible for sugarcane varieties released in the last decades [1], although some efforts exploiting genetic modification have been carried out [9–13]. It is worth noting that, to the best of our knowledge, no transgenic line has been commercially released yet [14, 15].

Sugarcane was brought to Brazil in 1531 and despite the enormous agronomic work to generate more productive lines, the understanding of the sugarcane physiology, especially in relation to its photosynthetic performance, is still lacking [16]. Modern cultivars have been selected mainly for characteristics based on nutritional demand and resistance to biotic and abiotic stresses, and not for photosynthetic activity, as has being done for the wheat and rice breeding programs [17]. Recent studies indicate a positive correlation among photosynthesis, crop yield and biomass production, suggesting that increasing photosynthesis is a potential way to enhance sugarcane productivity [16, 18–20].

In order to increase and/or manipulate sugarcane photosynthesis we must first understand the regulatory processes involved in C_4_ biochemistry [21]. C_4_-type photosynthetic metabolism is more efficient than the C_3_-type due to some physiological, biochemical and anatomical specific features [18, 22]. Species with C_4_ metabolism appeared independently at least 45 times during land plant evolution [23] in a minimum of 62 monocot and dicot plant species around 30 and 15 million years ago, suggesting that relatively simple genetic and regulatory mechanisms can drive the conversion of a C_3_ phenotype into C_4_ [24]. Nevertheless, the mechanisms and regulatory players are not yet fully understood.

Besides environmental factors, photosynthesis is also controlled by the sink strength balancing source supply and sink demand [25–29]. In this context, the activity of enzymes and the expression of genes related to photosynthesis follow the source-sink relationship in sugarcane [26, 30–35]. Consequently, many studies evaluating gene expression have focused mainly on the sink, i.e., sugarcane culm development, providing insights into culm maturation and sucrose accumulation [36–40]. In contrast, only few studies have explored the physiological and biochemical causes of photosynthesis variation among cultivars and leaf types [16, 20, 41, 42].

Leaves of grasses are excellent systems to study the establishment of C_4_ photosynthesis because there is a cellular developmental gradient along the leaf blade, with the basal cells being undifferentiated and immature and the cells towards the tip becoming more mature and specialized [43–49]. The most studied C_4_ species is maize, with several works describing transcriptomic, proteomic and metabolomic differentiation along the leaf blade [43–48]. Leaf development has been studied in rice and compared to maize leaves in order to identify key regulatory components and metabolite profiles for C_4_ phenotype [48]. So far, there is no similar report on C_4_ species other than maize.

The molecular mechanisms for C_4_ leaf development are complex and this process in polyploid and aneuploid plants such as sugarcane remain largely unknown. In order to fill this gap, we have carried out a detailed study of sugarcane leaf to investigate molecular and physiological changes along the leaf blade segments representing different developmental stages. The approaches used in this work allowed us to identify differences in compounds and enzymes responsible for the variable photosynthetic capacity among leaf segments, and several relevant genes that might be subjected to further studies in order to increase photosynthesis and productivity.

## Results and discussion

### Leaf sampling

Despite being clonally propagated, sugarcane has a heterogeneous germination rate and initial vegetative growth. In order to standardize our experiment, we conducted a pilot study to evaluate the plant age at which the highest number of leaves would have the same length and also guarantee that all stalk reserves have been consumed in a way that the plants were solely dependent on photosynthesis for growth. The length distribution of leaf +1 (the first with the dewlap fully exposed) of 60 day-old plants is depicted on Figure S1 (Additional file 1). Leaves with length between 52.3 and 57 cm had the highest frequency (20 %) and this group was considered nearly homogenous and harvested for further analysis. The plant phenotype is shown in Figure S2 (Additional file 1). Additionally, to overcome the length difference, even in the homogeneous population, we collected leaf segments taking into account the proportion of each segment on each length and not a fixed distance from the base of the leaf. The segments were named Base “zero” (B0); Base (B); Middle (M) and Tip (T) (see Materials and Methods and Figure S3 – Additional file 1).

### Physiological and biochemical evaluations

In order to characterize sugarcane leaf segments, several physiological and biochemical evaluations were performed. We used intact leaves and were able to evaluate gas exchange and photochemistry only on the B and M segments. Due to technical limitations, it was not possible to carry out those measurements on the B0 and T (inability to fit the measuring chamber on the B0 segment without damaging the structure of the leaf basis and to cover the whole chamber area on the T segments). Full details on the methods are available on Additional file 1 and the results are shown in Figures S4 and S5 (Additional file 1).

The photosynthetic response to light revealed significant differences between leaf segments. The leaf B segment showed light saturation under lower light intensities as compared to the M (Figure S4A – Additional file 1), indicating differential photosynthetic capacity among these segments. On the other hand, the initial slope of the light response curve and the PSII yield (Figure S4B – Additional file 1) suggested that the photochemistry is similar between the B and M segments. Together, these data revealed a possible metabolic rather than photochemical limitation in carbon fixation among leaf segments. The apparent electron transport rate - ETR - (Figure S4C – Additional file 1) also supports this idea. The relation between ETR and photosynthesis -A - (Figure S4D – Additional file 1) represents the amount of electron transport through PSII per CO_2_ fixed and increased values suggest an activation of alternative electron sinks such as the Mehler reaction and nitrogen (N) metabolism [50].

**Fig. 1:**
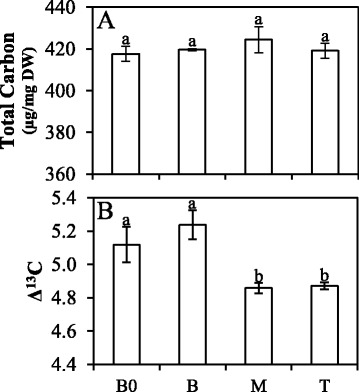
Total carbon quantification (**a**) and carbon isotope discrimination (**b**) in sugarcane leaf segments: Base “zero” (B0), Base (B), middle (M) and tip (T) sugarcane leaf segments. Letters indicate statistical significance using ANOVA followed by post hoc Student *t*-test (*n* = 5; *p* ≤ 0.05)

Photosynthetic response to CO_2_ was used to estimate some biochemical traits and indicated that the previously mentioned metabolic limitation was caused by differences in PEPcase activity and not RubisCO (Figure S5B and S5C – Additional file 1). Although total carbon content was similar between leaf segments (Fig. 1a), there were significant differences in carbon isotope discrimination among them (Fig. 1b). Carbon isotope discrimination has been used to characterize C_4_ photosynthetic responses in plants growing under diverse environments and stresses [51–56]. C_3_ plants have lower ∆^13^C than C_4_ plants, mainly because PEPcase has lower discrimination for ^13^C as compared to RubisCO [57]. Carbon isotope discrimination showed that the B0 and B segments presented higher ∆^13^C than M and T. The ∆^13^C variation in C_4_ plants is related to radiation intensity in maize, *Miscanthus giganthus* and *Flaveria bidentis*, which displayed higher ∆^13^C when cultivated under low light when compared to leaves exposed to high light [58–61]. This difference has been usually interpreted as a result of the leakiness or due to an inefficient C_4_ photosynthetic pathway. Nevertheless, the metabolic difference between leaf segments in sugarcane cannot be associated with leakiness that varied from 0.034 ± 0.013 to 0.031 ± 0.009 at B and M, respectively. In addition, the B segment presented a lower *k* when compared to the M segment (Figure S5C – Additional file 1), corroborating to the fact that the B has a less efficient C_4_ biochemistry than the M and T segments. Interestingly, while B0 and B had higher ∆^13^C than the rest of the leaf, the B segment showed lower photosynthetic rate (Figure S4A – Additional file 1) but similar PSII yield as compared to the M segment (Figure S4B – Additional file 1). This reinforces our interpretation that the C_4_ biochemical inefficiency in basal portions is higher than in the other parts of the leaf.

**Fig. 2:**
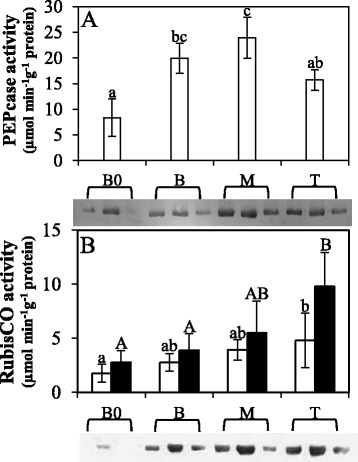
Changes in activity and protein amount of carboxylation enzymes in sugarcane leaf segments: Base “zero” (B0), Base (B), middle (M) and tip (T). (**a**) Phosphoenolpyruvate carboxylase (PEPcase). Letters indicate statistical significance using ANOVA followed by post hoc Student *t*-test (*n* = 4; *p* ≤ 0.05); (**b**) Ribulose-1,5-bisphosphate carboxylase/oxygenase (RubisCO). White bars represent initial activity and black bars total activity. Lower case letters and capital letters indicate statistical significance using ANOVA followed by post hoc Student *t*-test (*n* = 4; *p* ≤ 0.05) on initial and total activity, respectively. For the immunoblots the same amount of protein (100 μg) was loaded for each sample. Three independent biological replicates are shown for each segment

In order to investigate possible changes in PEPcase and RubisCO among the segments, immunoblotting and enzyme activity assays were performed. Although immunoblotting of leaf extracts showed some variation between biological replicates from the same segment, differences in PEPcase protein abundance were observed (Fig. 2a) with the highest amount being detected at the M segment. In addition, in vitro PEPcase activity was significantly higher at the M in comparison to the other segments (Fig. 2a), validating the in vivo *k* estimation (Figure S5C – Additional file 1). For RubisCO, the B0 segment was identified as the one with the lowest amount of both protein and activity (Fig. 2b). There was a tendency of increasing RubisCO activity along the leaf blade, but statistical significance was only detected between the B0 and T segments. The RubisCO activation state was also calculated as the ratio of initial activity to total activity but no substantial differences were detected among segments (0.63 ± 0.18, 0.70 ± 0.25, 0.75 ± 0.30, 0.54 ± 0.16 at B0, B, M and T, respectively), corroborating also with in vivo V_max_ estimation (Figure S5B – Additional file 1).

The number of stomata between leaf segments was similar when considering the adaxial and abaxial surfaces (Figure S6 – Additional file 1). This indicates that stomata density does not contribute to the variations observed at photosynthetic rates (Figure S5A). Although some studies reveal that stomatal density has a positive correlation with photosynthesis [62], differences in photosynthesis along leaf blades of several C_3_ and C_4_ grasses were not explained by stomata density [63].

**Fig. 3:**
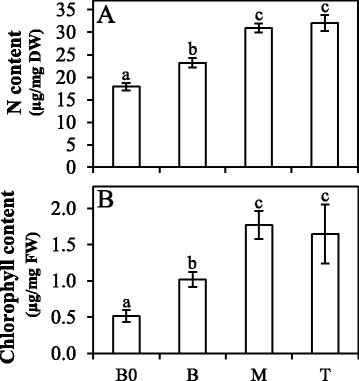
Total nitrogen quantification (**a**) and chlorophyll content (**b**) in sugarcane leaf segments: Base “zero” (B0), Base (B), middle (M) and tip (T). Letters indicate statistical significance using ANOVA followed by post hoc Student *t*-test (*n* = 5; *p* ≤ 0.05)

N content increased along the leaf length and was higher at the M and T segments (Fig. 3a). These results are very similar to those found in the plant canopy, where the bottom leaves have lower N concentration than upper ones due to variations in light availability [16, 64]. However, N investment on photosynthetic machinery was similar, with photosynthetic nitrogen-use efficiency (PNUE) varying between 1.08 ± 0.17 mol mol^-1^ h^-1^ at the B segment and 1.09 ± 0.14 mol mol^-1^ h^-1^ at the M segment. Chlorophyll concentration was also higher on the M and T segments in comparison to the B0 and B (Fig. 3b), but those differences were not sufficient to bring about changes in photochemical activity among the leaf segments (Figure S4B and S4C – Additional file 1).

The quantification of soluble sugars revealed a clear tendency of higher glucose, fructose and sucrose concentrations at the T when compared to the other segments (Table 1). Although the evaluated photosynthetic parameters showed significant differences between the B and M segments, they were not contrasting in terms of sugar content, the end products of photosynthesis. However, one should consider that sugar concentration in leaves is affected not only by current photosynthesis but also by its dynamic of consumption and exportation. Interestingly, metabolite profiling in fifteen 1 cm-long maize leaf segments showed that glucose and fructose levels were higher in regions close to the base and decreased along the leaf blade towards the tip. Sucrose, in contrast, presented a distinct behavior, with higher levels in segments close to the base and tip and lower levels in the middle regions [48]. Myo-inositol showed a clear gradient of accumulation from B0 to the T segment (Table 1). Myo-inositol has a central role in plant metabolism and can be used as precursor for the synthesis of phosphatidylinositol, compatible solutes (such as raffinose-family oligosaccharides) and cell wall polysaccharides [65]. In maize leaves, myo-inositol levels present the same trend observed in sugarcane leaves [48]. Together, our results suggest that the distribution of sugars along the sugarcane leaf is distinct from maize. However, any comparison between the two plant species must be regarded with caution. First, the age of the plants in the two studies are very different: while Wang et al. [48] have analyzed nine days-old maize leaves, the sugarcane plants in this study were two-months old. Second, the authors used the third leaf (from bottom to top) for maize, while for sugarcane we used the first leaf (from top to bottom) with the dewlap fully exposed.

**Table 1:** Quantification of soluble sugars

Segment/Sugar	sucrose	glucose	fructose	myo-inositol
Base “0”	15.05 ± 2.01^a^	0.53 ± 0.21^a^	0.25 ± 0.06^a^	165.93 ± 18.28^a^
Base	16.93 ± 6.15^a^	0.78 ± 0.09^a^	0.11 ± 0.05^a^	258.76 ± 25.69^b^
Middle	24.03 ± 7.63^ab^	1.69 ± 0.66^a^	0.37 ± 0.18^a^	439.48 ± 37.87^c^
Tip	29.99 ± 6.84^b^	4.50 ± 1.13^b^	2.69 ± 0.95^b^	953.45 ± 62.33^d^

Values shown are mean ± SD (*n* = 5) and referred to μmol g^-1^ FW, except for myo-inositol (nmol g^-1^ FW). Letters indicate statistical significant difference between leaf segments using ANOVA followed by post hoc Student *t*-test (*p* ≤ 0.05)

### Transcriptional profiling

#### RNA-seq *de novo* transcript assembly and annotation

The mRNAs from the segments (B0, B, M and T) of four individuals were sequenced and on average 33.8 million paired-end 100-bp strand-specific reads were obtained per segment and per individual (after quality trimming of the reads), with a total of 380 million high quality reads (after removal of contaminants, ribosomal RNA, mitochondrial and plastid reads). High quality reads were assembled with Trinity (version r20140717) as described in Methods. These data comprise 250,035 correctly oriented transcripts or contigs that were kept for further analysis, as they appear to originate from viridiplantae or did not had any hits to nucleotide sequences in the NCBI databases (Additional file 2). These 250,035 contigs were grouped into 135,481 loosely defined genes that could represent paralogous copies of the genes or copies from the homologous genomes. The average contig length was 878 bp with approximately 28 % of the contigs over 1Kbp long, and the smallest contig with 283 bp; 27.3 % (68,367) of the assembled contigs appear to code for proteins. 132,665 (53 %) contigs were annotated with Trinotate and 64,813 of these had Gene Ontology (GO) terms assigned (Additional file 3). The full set of *de novo* assembled contigs was compared to the full set of *Sorghum bicolor* proteins and transcripts (v2.1 Phytozome), using transrate (v1.0.1). On one hand, around 31 % of the sugarcane contigs had a high confidence predicted homologue (Conditional Reciprocal Best BLAST, CRBB) in *S. bicolor*, when compared to the sorghum proteins, and 40 % when compared to the sorghum transcripts. On the other hand, 60 % of the sorghum proteins and 65 % of the sorghum transcripts have CRBBs in sugarcane. We further inspected our transcriptome assembly using CEGMA, which has a set of 248 highly conserved eukaryotic genes that are usually present as single copy genes in many species. We were able to detect 72.18 % of these genes as complete proteins or 77.82 % when considering partial hits, with 3.63 copies of each gene on average. We have assigned 7,270 contigs into 333 KEGG pathways (Additional file 4) and identified 2,889 contigs belonging to 72 transcription associated families, i.e., transcription factors or other transcriptional regulators, by applying the procedure described in Perez-Rodriguez et al. [66] (Additional file 5). We also identified 38,399 groups of orthologous genes between the grasses Saccharum spp. (*de novo* assembled transcriptome), *Oryza sativa*, *Zea mays*, *Sorghum bicolor* and *Setaria italica* (all protein datasets were downloaded from Phytozome) using OrthoMCL (inflation value 1.5); 10,288 groups of orthologous genes are shared between the five species, more importantly 15,840 groups of orthologues are shared between sugarcane and any of the other grasses representing 36,629 sugarcane transcripts, with 7,339 groups of orthologues present exclusively in sugarcane (Figure S7- Additional file 1). These results show the overlap in protein space between these species, and highlights that our *de novo* sugarcane transcriptome assembly recovered a large proportion of the genes that are present in grasses (Additional file 6).

### Analysis of transcript abundance and differential expression analyses

**Fig. 4:**
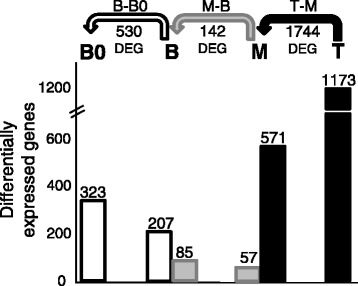
Representation of the RNA-seq contrasts between segments. Each segment was compared against the previous one (basal/distal length) originating three contrast: Base - Base "zero" (B-B0); Middle – Base (M-B); Tip – Middle (T-M). The number of differentially expressed genes (DEG) is depicted under the arrows representing the contrasts. Number of genes overexpressed on each segment considering different contrasts is shown above the graphic bars

**Fig. 5:**
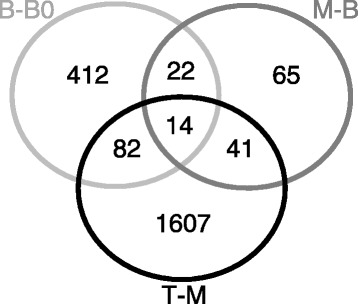
Venn diagram showing the overlap and exclusiveness of genes from each contrast: Base - Base "zero" (B-B0); Middle – Base (M-B); Tip – Middle (T-M)

In this study, we aimed to generate a transcriptome resource to evaluate the developmental dynamics along the sugarcane leaf. For that, we estimated transcript abundances using eXpress, read counts were analyzed in edgeR and normalized using the “trimmed mean of M values” (TMM) method [67]. Comparisons between segments were performed subtracting distal from basal segments originating the three orthogonal contrasts: Base *vs*. Base "zero" (B-B0 ); Middle *vs*. Base (M-B); Tip *vs*. Middle (T-M) (Tables S1–S3 – Additional file 7). For this analysis we have summarized the read counts at the level of genes as defined above (R code and read counts per gene per sample are available as Additional file 8). The representation of the contrasts and the number of differentially expressed (DE) genes are depicted on Fig. 4. The Venn diagram shows that the leaf undergoes a drastic transcriptional rearrangement along the developmental gradient (Fig. 5). This is even more evident when considering the number of DE genes shared among the contrasts (Fig. 5). Only 14 genes were present in all contrasts (Fig. 5 and Table 2) and almost 72 % of all DE genes were present only in the T-M contrast (Fig. 5). This is in agreement with Majeran et al. [45] and Pick et al. [49], who found higher the amount of transcripts and proteins toward the tip of maize leaves. We observed the same pattern on sugarcane, not only on transcripts number, but also by extracting and quantifying protein content (Figure S8 – Additional file 1).

**Table 2:** Genes identified in all contrasts [Base -  Base "zero" (B - B0); Middle – Base (M-B); Tip – Middle (T-M)]

	Contrast				
Gene ID	B-B0	M-B	P-M	Annotation	Validation
SP803280_c109776_g3	2.48	1.70	2.07	Transcription factor TCP5	0.98
SP803280_c88088_g1	-1.10	-2.11	-1.71	N/I	0.26
SP803280_c108434_g2	-1.50	-1.60	-1.90	Protein trichome birefringence	0.27
SP803280_c113688_g1	-1.51	-2.24	-1.49	4-hydroxyphenylacetaldehyde oxime monooxygenase	0.96
SP803280_c103567_g1	-1.58	-1.89	-1.54	N/I	0.62
SP803280_c110116_g1	-1.62	-2.01	2.11	Cysteine-rich repeat secretory protein 38	0.56
SP803280_c99583_g1	-1.92	-2.74	-3.89	N/I	0.94
SP803280_c109581_g5	-2.01	-2.96	2.13	Methylsterol monooxygenase 1-2	0.71
SP803280_c87779_g1	-2.33	-2.12	-2.12	N/I	0.42
SP803280_c81421_g1	-2.37	-1.81	-2.87	Cell wall / vacuolar inhibitor of fructosidase 2	0.56
SP803280_c114207_g1	-2.56	-2.83	-2.88	Cycloartenol-C-24-methyltransferase 1	0.79
SP803280_c100769_g2	-2.66	-3.24	-3.62	N/I	0.99
SP803280_c70793_g2	-2.74	-2.01	-1.97	N/I	0.87
SP803280_c116667_g1	-3.61	5.54	2.25	Naringenin,2-oxoglutarate 3-dioxygenase	0.69

The number bellow each contrast indicates the log Fold Change in the respective contrast

Validation column indicates the average coefficient of determination (R^2^) between logCPM (from RNA-seq data) and logΔCt (qRT-PCR data)

Considering the 14 differentially expressed genes that are present in all contrasts, we were able to identify four expression patterns along the leaf developmental gradient (Figure S9 – Additional file 1). The first pattern (Figure S9A – Additional file 1) is composed by the transcriptional factor TCP5 (SP803280_c109776_g3) that monotonically increased its expression from B0 to T segments. Members of this family are involved with leaf morphogenesis and differentiation [68–70], arrest of cell division [71], leaf elongation [72] and auxin response [73]. To date, there is no information on the role of TCP5 in C_4_ plants and its expression profile in our dataset suggests that this gene can be an important player during leaf development.

Ten genes from the second pattern (Figure S9B – Additional file 1) presented an opposite behavior, with high expression at B0 segment and decreasing expression towards the T segment. Among them, we can point out the trichrome birefringence gene (SP803280_c108434_g2), which is important for o-acetylation of cell walls required for cellulose biosynthesis [74–76], and the gene encoding a 4-hydroxyphenylacetaldehyde oxime monooxygenase (SP803280_c113688_g1) involved in the production of a cyanogenic glycoside called dhurrin [77, 78]. This compound has been related to drought tolerance in sorghum [79] and to biotic stress response [80]. Interestingly, dhurrin can also be regarded as an N storage molecule that peaks on early development stages in sorghum [81]. Genes involved in carbohydrate and sterol metabolism were also classified into this pattern: the cell wall and vacuolar inhibitor of fructosidase 2 (SP802180_c81421_g1) responsible for post-transcriptional silencing of fructosidase activity and important in the development of photosynthetic apparatus, stress response and sugar signaling [82, 83]; and the cycloartenol-C-24-methyltranferase 1 (SP803280_c114207_g1- also known as SMT1) that catalyzes the initial step in biosynthesis of sterol, a class of compound with several regulatory roles in plant development [84]. SP803280_c87779_g1 code for an invertase/pectin methylesterase inhibitor ortholog of LOC_Os08g01670.1 and LOC_Os12g18560.1 in rice [85], and thus might be involved in the remodeling of the plant cell wall. SP803280_c100769_g2, coding for a dirigent protein ortholog to LOC_Os01g24960.1 and LOC_Os01g25030.1 in rice, involved in lignin biosynthesis [86]. An additional gene SP803280_c103567_g1 with orthologues in setaria, maize and sorghum, but not in rice is of unknown function, while SP803280_c88088_g1 and SP803280_c99583_g1 do not appear to code for proteins. In summary, genes that presented the second pattern are involved in early developmental processes and cell wall modification, corroborating with their expression on the most basal segment.

The pattern 3 was comprised by genes with lower expression at the M segment (Figure S9C – Additional file 1), like those encoding methylsterol monooxygenase 1–2 (SP803280_c109581_g5) and cysteine-rich repeat secretory protein 38 (SP803280_c110116_g1). Methylsterol monooxygenase is involved in sterol metabolism [87], important for membrane fluidity and membrane interaction with proteins and lipids [88, 89]. Arabidopsis has more than 100 genes coding for cysteine-rich repeat proteins making them one of the largest gene families. However, their role on plant metabolism is still to revealed [90].

Pattern 4 (Figure S9D – Additional file 1) is characterized by lower expression at B segment and had only one gene, encoding a naringenin, 2-oxoglutarate 3-dioxygenase (SP803280_c116667_g1). This protein participates in the flavonoids biosynthesis [91], important for UV protection, defense against pathogens and pests, regulation of auxin transport and pigmentation [92].

All those 14 genes were also used to validate RNA-seq data by qRT-PCR using three biological replicates (different from those used for the RNA-seq experiment). The average coefficient of determination (R^2^) between logCPM and logΔCt was 0.70 (Table 2). Genes with low expression values (SP803280_c103567_g1, SP803280_c108434_g2, SP803280_c110116_g1, SP803280_c116667_g1, SP803280_c70793_g2) had the lowest R^2^ correlation as reported before for genes with similar expression levels [93–95]. However, it is worthwhile mentioning that despite these low R^2^ values, the general expression profile obtained by qRT-PCR resembled those obtained by RNASeq. We cannot ignore the pitfalls and artefacts of each technique, but one possible explanation might be the fact that we have a *de novo* transcriptome assembly of a crop with a complex polyploid genome that has not been sequenced yet. This represents an extra layer of difficulty when designing primers for qRT-PCR, as it is not possible to distinguish between all the alleles and paralogues, increasing the variability of qRT-PCR data.

**Fig. 6:**
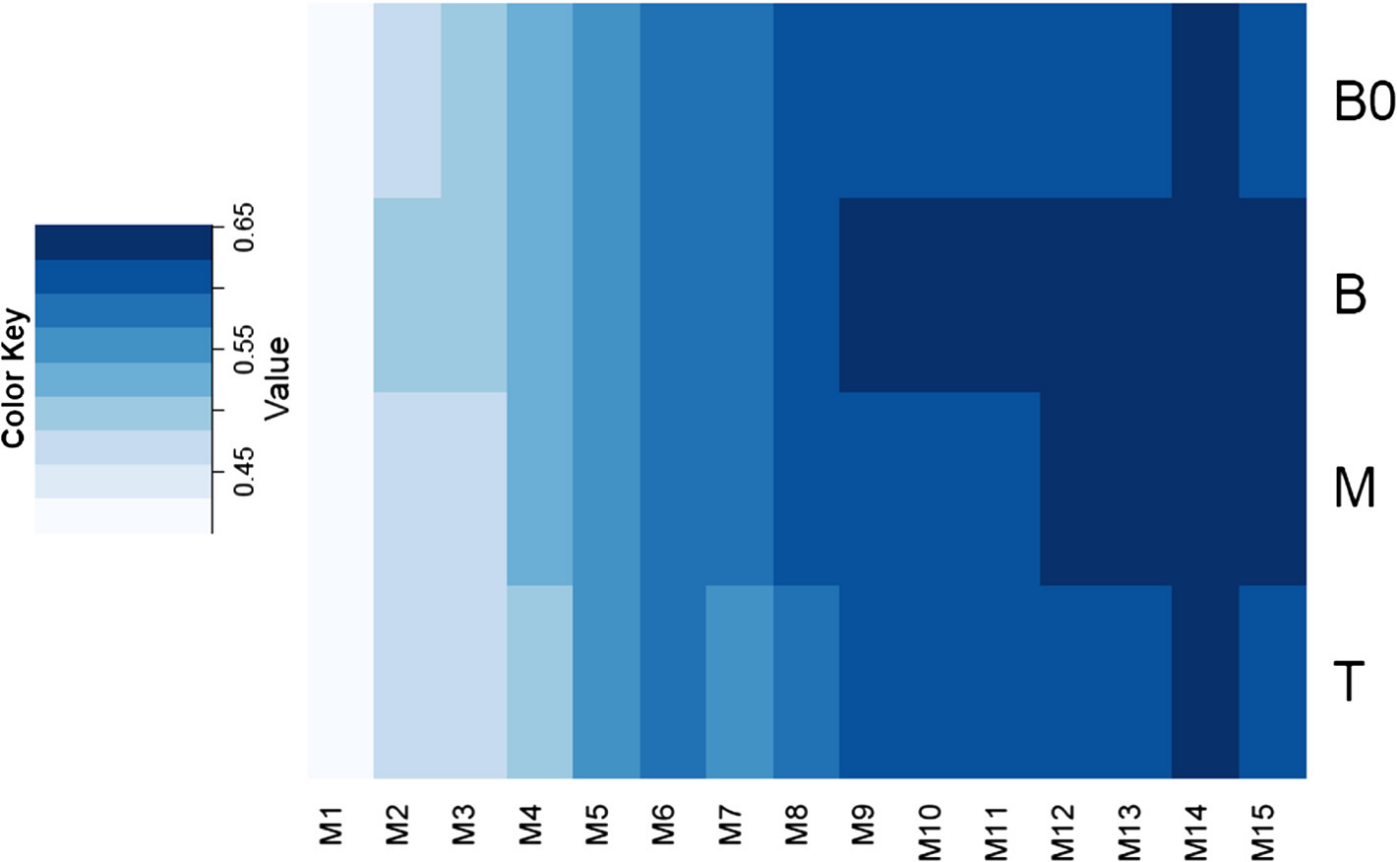
Spearman correlation between fifteen segments along developmental gradient of maize leaves (M1 to M15 - published by [48]) and the four sugarcane leaf segments (B0, B, M and T - this study)

We were also interested in comparing the transcriptional profile of sugarcane (this study) to the one observed in another C_4_ species, maize (recently published by Wang et al [48]). We compared the expression of over 2,390 one-to-one orthologous genes between sugarcane and maize, identified by OrthoMCL (Fig. 6), in a similar fashion as described by Wang et al. [48] (Data matrices and R code are available as Additional file 9). The expression profiles during leaf development between these two species were substantially different as the whole developmental gradient of the sugarcane leaf fits better into the distal half of the maize leaf (Spearman correlation coefficient higher than 0.6 – Fig. 6).

### Enrichment of gene ontology terms

A GO enrichment analysis was performed by categorizing differentially regulated genes into GO biological processes (Tables S4-S9 – Additional file 10). Each contrast was divided into two gene lists in order to evaluate the enrichment of biological processes. For instance, the contrast B-B0 was separated into positive log FC – genes more expressed at the B segment – and negative log FC – genes more expressed at the B0 segment). Considering the contrast B-B0, the most significant biological processes in B0 segment were those involved with cell wall organization and biosynthesis (e.g. GO IDs 71669, 71554, 9664, 9832 and 42546). It is noteworthy that wax metabolic processes (GO ID 10166), anatomical structure development (GO ID 48856), developmental process (GO ID 32502) and leaf formation (GO ID 10338) were also enriched terms in this segment (Table S4 – Additional file 10). In the B segment, the most significant GO terms were flavonoid biosynthetic and metabolic processes (GO ID 9813 and 9812), redox process (GO ID 55114), cellular response to high light (GO ID 71486) and lipid metabolic process (GO ID 6629) (Table S5 – Additional file 10).

The GO enrichment analysis indicated that in the contrast M-B, genes related to DNA modification (GO IDs 6334, 34728, 31497, 71824, 65004, 6333, 16126, 6323) and N metabolism (GO ID 10243 and 1901698) were more expressed in the B segment (Table S6 – Additional file 10). In the M segment, only two GO IDs were enriched (9813, 9812), both related to flavonoid metabolism (Table S7 – Additional file 10).

Interestingly, the largest amount of DE genes was found between the M and T, the segments that demonstrated the most similar behavior according to the physiological data. On the other hand, the M and B showed the smallest amount of DE genes in spite of their differences in physiological behavior, especially photosynthetic capacity, carbon isotope discrimination and N content. Considering the T-M contrast, the most overrepresented GO IDs in the M were those related to photosynthesis and redox processes (15979, 55114, 19684) (Table S8 – Additional file 10). The T segment presented the highest number of enriched terms related to amino sugar catabolic process (GO ID 46348), ion transport (GO ID 6811), transmembrane transport (GO ID 55085), zinc ion transport (GO ID 6829), cation transport (GO ID 6812) and anion transport (GO ID 6820) (Table S9 – Additional file 10).

### Biochemical pathways

#### Photosynthesis-related genes

It is already known that the C_4_ biochemical pathway is transcriptionally regulated [96, 97] under developmental [98] and light [99] control. In our study, only one NADP-dependent malic enzyme (SP803280_c89172_g1) was differentially expressed in the B0-B contrast (Table S1 – Additional file 7). This indicates that CO_2_ concentrating mechanism is still under development in the B0 segment, which is less exposed to light than the other ones. Two phosphoenolpyruvate carboxykinases (PEPCK - SP803280_c92961_g1 and SP803280_c92945_g1) were more expressed at the B segment (contrast M-B, Table S2 – Additional file 7). This enzyme has been reported as an important regulator of the aspartate metabolism in bundle sheath cells where aspartate is decarboxylated to PEP by PEPCK and CO_2_ supplied to the Calvin-Benson-Brassham cycle [100].

Nevertheless, the contrast T-M showed the highest amount of photosynthesis related genes (Table 3), and the majority of them was more expressed at the M segment such as enzymes of the Calvin-Benson-Brassham cycle and proteins associated with chlorophyll and photosystems. Interestingly, the expression of four PEPcase genes was higher at the T segment in comparison to the M segment. This finding corroborates with the qPCR data reported by Li et al. [44], in which the transcript level of one PEPcase increasing towards the tip of maize leaves. However, our immunoblotting and enzymatic assays revealed greater content and activity at the M segment (Fig. 2) and decrease in both activity and content at the T. Additionally, PEPcase is known to undergo post-transcriptional and post-translational modifications [98, 101, 102] and a recent study demonstrated that the Spearman rank correlation (R_S_) between mRNA and protein abundance for genes related to photosynthesis on different sections of maize leaf was 0.581 on average [103], indicating that PEPcase expression profile may not be an indication of more photosynthetically active tissue. One NADP-dependent malic enzyme was also more expressed at the T segment (SP803280_c89172_g1), corroborating with other reports that indicated its increase towards the tip [104, 105].

**Table 3:** Photosynthesis related genes identified on each contrast

GeneID	Annotation	LogFC
Contrast B-B0		
SP803280_c89172_g1	NADP-dependent malic enzyme	-1.28
SP803280_c102133_g1	Fructose-bisphosphate aldolase, chloroplastic	1.36
Contrast M-B		
SP803280_c92945_g1	Phosphoenolpyruvate carboxylase kinase 1	-1.70
SP803280_c92961_g1	Phosphoenolpyruvate carboxylase kinase 1	-2.16
Contrast T-M		
SP803280_c110449_g1	Ribose-5-phosphate isomerase 4, chloroplastic	-3.87
SP803280_c67360_g1	Photosystem II reaction center W protein, chloroplastic	-3.62
SP803280_c95106_g1	Chlorophyll a-b binding protein, chloroplastic	-3.37
SP803280_c92288_g1	NADP-dependent malic enzyme, chloroplastic	-2.87
SP803280_c102650_g1	Magnesium-chelatase subunit ChlI, chloroplastic	-2.38
SP803280_c99238_g1	Chlorophyll a-b binding protein CP24 10B, chloroplastic	-2.33
SP803280_c104535_g4	Glyceraldehyde-3-phosphate dehydrogenase GAPB, chloroplastic	-2.32
SP803280_c103134_g1	Phosphoenolpyruvate carboxylase 3	-2.28
SP803280_c80781_g1	Photosystem I reaction center subunit VI, chloroplastic	-2.27
SP803280_c97299_g1	Carbonic anhydrase, chloroplastic	-2.19
SP803280_c92767_g1	Ribulose bisphosphate carboxylase small chain, chloroplastic	-2.17
SP803280_c92767_g1	Ribulose bisphosphate carboxylase small chain, chloroplastic	-2.17
SP803280_c96992_g1	Photosystem I reaction center subunit XI, chloroplastic	-2.04
SP803280_c94064_g1	Photosystem II reaction center PSB28 protein, chloroplastic	-2.03
SP803280_c86157_g1	Chlorophyll a-b binding protein 7, chloroplastic	-1.99
SP803280_c104447_g1	Photosystem II core complex proteins psbY, chloroplastic	-1.99
SP803280_c88331_g1	Ferredoxin-thioredoxin reductase, variable chain	-1.74
SP803280_c87990_g4	ATP synthase delta chain, chloroplastic	-1.73
SP803280_c100740_g1	Photosystem II repair protein PSB27-H1, chloroplastic	-1.65
SP803280_c89062_g1	Photosystem I reaction center subunit III, chloroplastic	-1.52
SP803280_c95984_g2	Photosystem II core complex proteins psbY, chloroplastic	-0.98
SP803280_c97299_g2	Carbonic anhydrase, chloroplastic	-0.73
SP803280_c20755_g1	Ferredoxin--NADP reductase, leaf isozyme, chloroplastic	-0.63
SP803280_c89172_g1	NADP-dependent malic enzyme	1.18
SP803280_c108467_g2	Phosphoenolpyruvate carboxylase 1	1.26
SP803280_c57883_g1	Phosphoenolpyruvate carboxylase 1	1.29
SP803280_c25925_g1	Phosphoenolpyruvate carboxylase 1	1.54
SP803280_c89145_g1	Phosphoenolpyruvate carboxylase 1	1.88

LogFC: Log Fold Change. Positive LogFC indicates more expression at the more basal segment of the contrast; negative LogFC indicates more expression at the most distal segment of the contrast

The gene encoding a glyceraldehyde-3-phosphate dehydrogenase (SP803280_c104535_g4) was expressed at the T segment and the expression of the Arabidopsis homologue is increased under several stress conditions such as heat, anoxia and high sucrose concentration [106]. In fact, the T segment has lower leaf water content (Figure S10 - Additional file 1), which may lead to stress responses similar to oxidative stress in a similar fashion described by Pick et al. [49] for maize leaf tips. Accordingly, many genes related to oxidative stress were expressed at the T segment.

### Sugars

Genes related to carbohydrate metabolism are depicted on Table 4. Analysis of the B-B0 contrast revealed that two genes (SP803280_c111302_g1 and SP803280_c117830_g1) more expressed at the B0 segments encoded proteins involved in the synthesis of trehalose-6-phosphate (Tre6P), the phosphorylated intermediate of the non-reducing sugar trehalose. Tre6P is considered a signal of sucrose availability and acts to maintain sucrose concentration within a proper range [107, 108]. We have not quantified the levels of Tre6P in our experiments, but sucrose content was similar at B0, B and M segments (Table 1). The above-mentioned genes encode Tre6P synthase class II proteins (Figure S11 – Additional file 1), which in *Arabidopsis thaliana* do not have catalytic activity and are of unknown function [109]. In addition, a transcript related to sucrose metabolism (sucrose synthase 2 - SP803280_c114621_g2) was also induced in the B0 segment.

**Table 4:** Sugar-related genes identified on the contrast Tip - Middle (T-M)

GeneID	Annotation	LogFC
SP803280_c96835_g1	Purple acid phosphatase 2	-3.23
SP803280_c109484_g1	Alkaline/neutral invertase CINV1	-2.91
SP803280_c115744_g2	Phosphoinositide phospholipase C 4	-2.67
SP803280_c106015_g1	Fructose-bisphosphate aldolase, cytoplasmic isozyme	-2.36
SP803280_c111302_g1	Alpha,alpha-trehalose-phosphate synthase [UDP-forming] 8	-1.48
SP803280_c114621_g2	Sucrose synthase 2	-1.29
SP803280_c117830_g1	Alpha,alpha-trehalose-phosphate synthase [UDP-forming] 5	-1.17
SP803280_c113920_g1	Phospholipase D delta	-0.93
SP803280_c117255_g1	Glucose-6-phosphate isomerase, cytosolic	-0.64
SP803280_c95757_g2	Pyrophosphate-fructose 6-phosphate 1-phosphotransferase subunit alpha	0.57
SP803280_c118055_g1	Alpha-glucosidase	0.59
SP803280_c85603_g1	Phosphoglycerate kinase, cytosolic	0.59
SP803280_c107723_g2	Beta-fructofuranosidase, insoluble isoenzyme 4	0.61
SP803280_c111639_g5	Phosphoglycerate mutase GpmB	0.68
SP803280_c114046_g3	Fructokinase-1	0.72
SP803280_c87942_g1	Alkaline/neutral invertase CINV2	0.79
SP803280_c98160_g1	Sucrose synthase 1	0.85
SP803280_c94862_g2	Purple acid phosphatase 2	0.94
SP803280_c105243_g1	UDP-glucose 6-dehydrogenase 4	1.02
SP803280_c101982_g1	Inositol-3-phosphate synthase	1.09
SP803280_c99372_g1	Purple acid phosphatase 2	1.15
SP803280_c94306_g1	Plastidial pyruvate kinase 2	1.16
SP803280_c100721_g1	Alkaline/neutral invertase CINV2	1.26
SP803280_c114269_g1	Soluble starch synthase 3, chloroplastic/amyloplastic	1.29
SP803280_c107054_g1	Beta-fructofuranosidase 1	1.38
SP803280_c101458_g2	Aldose 1-epimerase	1.39
SP803280_c109150_g1	Beta-fructofuranosidase 1	2.63

LogFC: Log Fold Change. Positive LogFC indicates more expression at the T segment; negative LogFC indicates more expression at the M segment

In the M-B contrast only one gene encoding an invertase (SP803280_c100721_g1) was more expressed in the M segment. The vast majority of DE genes related to sugars was observed in the contrast T-M (Table 4). Several genes involved in starch and sucrose metabolism and interconversion of hexoses-phosphate were upregulated in the T segment. In addition, a gene coding for an inositol-3-phosphate synthase (SP803280_c101982_g1), a key enzyme in the conversion of glucose to myo-inositol [110], was 2-fold more expressed in the T relative to M. Such finding is in agreement with the concentration of this sugar alcohol at the T segment (Table 1). Two purple acid phosphatase 2 genes (SP803280_c99372_g1 and SP803280_c94862_g2), responsible for the dephosphorylation of myo-inositol hexakisphosphate (a phosphorus storage molecule) have been upregulated in the T segment whereas only one gene (SP803280_c96835_g1) was more expressed in the M segment. Furthermore, phosphoinositide phospholipase C4 (SP803280_c115744_g2) and phospholipase D (SP803280_c113920_g1) which participate on inositol signaling [111] were more expressed in the M segment.

Although many changes in transcripts related to enzymes of the sugar metabolism have been noticed, we could not directly link transcript abundance to the quantified sugars in most cases. A possible explanation is that other factors are influencing protein activity such as translation efficiency, protein assembly and degradation [112].

### Cell wall biosynthesis and cell growth

Several cell wall related genes were DE in the tested contrasts (Table S10 – Additional file 11), indicating cell wall modification along the leaf developmental gradient. Among all identified genes, the pH-dependent cell wall loosening proteins known as expansins [113] were more expressed in the B0 (contrast B-B0) and T (contrast T-M) segments, indicating that the extreme opposite sides of the sugarcane leaf are under cell wall modification when compared to the other adjacent segments (Figure S12 – Additional file 1). COBRA genes have an important role in cellulose synthesis [114], and, together with cellulose synthase genes, were more expressed in the B0 and T segments. In the most basal segment (i.e. B0), cellulose synthesis is expected to be part of secondary cell walls and structure of vascular system, whereas in the T segment we assume cell wall modifications due to senescence.

Suberin is a heteropolymer formed by lipid and phenolic compounds [115, 116] deposited in the bundle sheath cells [117] and may serve as a physical barrier to avoid CO_2_ leakiness to the mesophyll cells [118, 119]. Although suberin biosynthetic and regulatory pathways have not been defined for monocots yet [48], some reports identified few genes putatively involved in those processes [48, 117, 120]. Suberin genes identified in all contrasts are listed at Table S11 (Additional file 11). NAC and MYB transcriptional family members might be involved in regulating secondary cell wall and suberin biosynthesis [121–123]. Most of these genes were up regulated in the B0 (contrast B-B0 – Table S1 – Additional file 10) and in B (contrast M-B - Table S2 – Additional file 7) segments and the contrast T-M (Table S3 – Additional file 7) presented different expression patterns, indicating that suberization started at the basal portions of leaves before they become fully photosynthetically active.

### Transcription factors

Our analysis identified 1,057 genes belonging to 72 transcription factors or other transcriptional regulator families. Enrichment analysis of each contrast (same as described previously in GO analysis) indicated that very few families, if any, were enriched in each segment. However, we could identify several transcriptional factors differentially expressed among segments (Tables S1–S3 – Additional file 7).

The ARF transcriptional factor family and the AUX/IAA family of other transcriptional regulators are enriched at the B0 segment (Table S12 – Additional file 12). They are involved in the regulation of auxin responsive genes and have several roles in plant development [124], including leaf vascular differentiation [125]. Several AUX/IAA and ARF genes are more expressed at the B0 (SP803280_c113162_g1, SP803280_c111058_g1, SP803280_c94112_g1, SP803280_c114984_g4) when considering the contrast B-B0. AUX/IAA and ARF are also associated with stomatal development [126]. In maize, 31 members of this family have been identified [127]. We have identified 29 genes belonging to these two families in our RNA-seq dataset, but we are not able to state how many more members there are in sugarcane due the lack of a complete sugarcane genome sequence.

Members of bHLH and MYB families, involved in leaf development, were also more expressed in the B0 segment (Table S12 – Additional file 12). Even though MYB transcriptional factors are already known to regulate leaf development in tobacco [128] and tomato [129], bHLH might play a role in controlling the abaxial-adaxial polarity [130] and stomatal development [131].

In the leaf basis, the TCP family was enriched (Table S13 – Additional file 12) and more expressed at the contrast B-B0 (SP803280_c109776_g3). This family has an important role in developmental processes by regulating cell division in vegetative and reproductive structures. In Arabidopsis, TCP15 modulates cell cycle genes [132] and is involved in leaf development and regulation of auxin and cytokinin homeostasis [133–135].

No transcription factor family was enriched in the B (M-B contrast) and M (both M-B and T-M contrasts) segments (Tables S14–S16 - Additional file 12). On the other hand, the T segment was enriched in genes from the NAC family (Table S17 – Additional file 12), which has been associated with senescence in some plant species [136–139].

### Nitrogen assimilation and metabolism

N assimilation genes were up-regulated at the B0 segment (Table S1 – Additional file 7). Accordingly, Wang et al. [48] have reported that the leaf basal portions are responsible for N assimilation. The nitrate transporter gene (SP803280_c104238_g2), characterized in the classes of membrane proteins and involved in nitrate transport [140], was upregulated in the B region. Furthermore, the N concentration at B0 was lower than the others. The main genes associated to N metabolism and assimilation were more expressed in the M and T segments (Table S3 – Additional file 7). The genes glutamate synthase (GOGAT; SP803280_c109031_g1) and glutamate dehydrogenase 2 (GDH; SP803280_c111059_g2), involved in glutamate biosynthesis from ammonium ions [141, 142] were upregulated in the M and T segments, respectively. The genes which participate in N metabolism from nitrate source, such as nitrate reductase (SP803280_c104238_g2; [143] and ferredoxin-nitrite reductase (SP803280_c107711_g3; [144] were upregulated in the T segment. These data suggest that N metabolism and its assimilation are modulated along sugarcane leaf and that these processes are concentrated in more mature regions of sugarcane leaves.

### Leaf senescence

Leaf senescence occurs naturally in the quiescent cells and its onset and progression are controlled by external and internal factors. Factors like age, hormone levels and reproductive growth cause differential gene expression, resulting in macromolecule degradation, such as proteins, lipids, pigments (chlorophyll *a* and *b*; carotenoids) and nucleic acids [145–147] followed by recycling and mobilization of nutrients [147]. According to the transcriptional profile of different leaf segments, some genes and transcription factors (WRKY and NAC family) associated positively with the senescence pathway [136–139, 148] were overexpressed in the T segment (Family: NAC, SP803280_c104996_g2) as revealed in the contrast T-M (Table S3 – Additional file 7). The gene for the blue copper-binding protein (SP803280_c94951_g1), considered a senescence associated gene - SAG - [149], had a significant increase in expression level in the T segment.

In the immature B0 segment, no differential expression of SAGs was noticed (Table S1 – Additional file 7). However, we found a significant expression of gene encodig E3 ubiquitin-protein ligase ATL41 (SP803280_c95253_g2) and E3 ubiquitin-protein ligase UPL5 (SP803280_c109558_g1). These enzymes catalyze polyubiquitination and regulate leaf senescence negatively through ubiquitination and subsequent degradation of WRKY53, a key transcription factor of leaf senescence [150, 151]. This result suggests that the basal segment is functional and mechanisms of senecence avoidence are active, as expected for a young and immature leaf portion.

There are several genes involved in the chlorophyll degradation pathway [152], and some of them were identified as more expressed in the T (contrast T-M, Table S3 – Additional file 7). A first step to chlorophyll degradation is the change of chlorophyll–apoprotein complex structure and subsequent enzymatic breakdown of complex constituents by stay green proteins [153, 154]. The gene that encodes the protein STAY GREEN (SP803280_c105792_g2) was more expressed in the T compared to the M segment, suggesting a possible beginning of senescence mechanism.

Other pigments, such as carotenoids, are also degraded during leaf senescence. The gene encoding the enzyme carotenoid 9,10(9’,10’)-cleavage dioxygenase (SP803280_c114734_g1) responsible for the cleavage of carotenoids [155] was also upregulated in the T segment. These results indicate that the leaf senescence process begins at the leaf tip and that chlorophyll and carotenoids degradation are associated [136]. Although our analysis did not indicate chlorophyll degradation (Fig. 3), it seems that the leaf tip, at the molecular level, presents indications of the onset of senescence.

The GO enrichment analysis also revealed GO terms associated with “aging” and “cell killing” overrepresented at the T segment (Table S9 – Additional file 10). Three 1-aminocyclopropane-1-carboxylate oxidase are on this list, responsible for an important step on ethylene production [156]. Ethylene is a gaseous phytohormone and has an important role on the onset and progression of senescence [157]. The inhibition of its perception or biosynthesis in tobacco and tomato caused delayed onset of leaf senescence and lower expression of SAGs, which was also found in Arabidopsis ethylene-insensitive mutants [158–161].

### Gene expression peaking at the middle section of the leaf

Besides describing the physiological and transcriptional variation in sugarcane leaves, we also aimed to identify genes associated with high photosynthetic activity. To achieve this goal, we evaluated, amongst all genes expressed on our transcriptome, those that peaked their expression at the M segment, region with the highest photosynthetic capacity (Fig. 2 and Figures S4 and S5 – Additional file 1). For that, we utilized the software TimeSearcher [162] using the Z-values based on FPKM (Figure S13 - Additional file 1). We then looked for genes that had a normalized expression value (*Z*-value) at the B0, B and T segments lower than the average normalized expression for the M segment (*Z*-value < 0). In such a way, we identified 986 genes with expression values higher at the M segment and 26 % of which having functional annotation (Table S18 - Additional file 13).

GO enrichment analysis indicated that some pathways were enriched in the M segment (Table S19 - Additional file 13), even though the number of genes on each pathway is small. Some of them are related to starch and sucrose metabolism (SP803280_c92108_g1; SP803280_c109193_g3; SP803280_c102083_g2; SP803280_c90189_g1), amino sugar and nucleotide sugar metabolism (SP803280_c102083_g2; SP803280_c90189_g1; SP803280_c110680_g1; SP803280_c106685_g3); N metabolism (SP803280_c45945_g1; SP803280_c93257_g1) and plant hormone signal transduction (SP803280_c82109_g1; SP803280_c99047_g1).

From the genes directly related to photosynthesis, only two RubisCO transcripts (SP803280_c90358_g1 and SP803280_c102172_g2) presented higher expression at the M segment. However, our *in vivo* (Figure S5A – Additional file 1) and *in vitro* activities (Fig. 2b), and immunoblotting analysis (Fig. 2b) indicated no differences between segments (except B0 that always had the lowest values). Intriguingly, none PEPcase transcript was identified in this analysis, contrasting with the physiological and biochemical assays (Fig. 2a and Figure S5A- Additional file 1). As mentioned before, PEPcase gene is known to suffer both post-transcriptional and post-translational modifications [98, 101, 102], which could justify such inconsistence between gene expression and protein activity and amount.

An aquaporin belonging to SIP1 family (SP803280_c107872_g1) and the Myb-related protein Zm38 (SP803280_c114598_g1) were also identified. The silencing of the aquaporin homolog in Arabidopsis has shown to decrease osmotic water permeability in mesophyll and bundle sheath cells, mesophyll CO_2_ conductance, photosynthesis, transpiration, and shoot biomass in Arabidopsis [163]. This indicates that aquaporins can contribute to the establishment of high photosynthetic rates on the middle of the leaf blade. MYB transcription factors are related to leaf development in tobacco [128] and to leaf and shoot architecture in tomato [129]. In addition, the myb-related protein Zm38 regulates negatively genes involved in anthocyanin biosynthesis [164] and also epidermal cell development [165, 166].

A protein that has strong similarity to the C-terminal region of the Mid domain of the Argonaute (AGO) protein MEL1 (SP803280_c113083_g2) was also present at the M. MEL1 is associated with small RNA-directed regulatory pathways [167] and some studies have already indicated the importance of small RNAs on abiotic stress response [168–170]. Even though it is not clear how many AGO genes there are in sugarcane genome, we were able to identify all the Rice AGO genes in four groups of orthologues (LeafDev_mcl15_5, LeafDev_mcl15_83, LeafDev_mcl15_342 and LeafDev_mcl15_6159; Additional file 6) and in each of them there is at least one sugarcane representative. However, the protein SP803280_c113083_g2 is not present in any of these groups of orthologous genes and may represent a novelty in sugarcane.

Developmental studies indicate the importance of AGO on rice sporogenesis [171], Arabidopsis female gamete formation [172], leaf, shoot and apical meristem development [173–175], stomata development [176], control of meiosis and DNA repair [177] and shoot meristem initiation in rice [178]. In Arabidopsis, the role of small RNAs on leaf development is well studied [179] and there is also evidence of the importance of miRNA in on leaf development of other species such as celery [180] and potato [181]. Although there is no direct evidence, our study in sugarcane and the work from Li et al. [44] in maize suggest that miRNAs must play an important role on leaf development of grasses, but it is still a topic to be explored.

## Conclusions

This is the first report evaluating sugarcane leaf segments representing different developmental stages and it has proven to be a valuable tool for investigating the genes that might be regulators of C_4_ syndrome. In our study we describe detailed physiological and biochemical analyses among leaf segments. We also have made use of the next generation sequencing technology RNA-seq to identify molecular differences along the leaf blade of sugarcane. In addition, we compared our data with previous work recently published for maize [48]. This analysis revealed that leaf development differs significantly between sugarcane and maize based on their transcriptional profile. Although this comparison was limited in some aspects, it indicates large differences between these two species pointed out the importance of studying other crops in order to acquire substantial novel knowledge to enable improvement of the photosynthetic capacity followed by increase in productivity.

## Methods

### Plant material and growth conditions

Sugarcane stalks from genotype SP80-3280 (*Saccharum* spp.) were kindly provided by Centro de Tecnologia Canavieira (CTC), Piracicaba SP, Brazil. Stalks were sectioned in order to have only one bud per section and germinated in trays containing vermiculite. After one month, plants with the same height were transferred to pots (3.5 L) containing pine-bark substrate and vermiculite (1:1). Plants were fertilized with N:P:K (10:10:10) every 15 days. The pots were watered daily and every week their distribution inside the greenhouse was randomized. After 60 days, leaf segments were collected between 10:00 h and 14:00 h. The first 2 cm of the base of the first leaf with exposed dewlap (leaf +1 following the system by van Dillewijn [182]) were collected. For the other segments, each leaf was measured and was divided into three equal thirds. Samples were taken considering 1 cm of each side of the middle of each third, except for the tip, according to Figure S3 – Additional file 1. The segments were named Base “zero” (B0); Base (B); Middle (M) and Tip (T). Samples were frozen in liquid nitrogen, ground to a fine powder and stored at -80 °C until further processing. Additionally, in order to improve the gene space coverage of the *de novo* transcriptome assembly we included a sample resulting from pooling different developmental stages and tissues from an additional plant of the same genotype, and the collected tissues were: leaf +1, shoot, and root; each after one and two months of growth under the same conditions described above.

### PEPcase and RubisCO activity and quantification

For determining the catalytic activity of the enzymes, aliquots of 50 mg FW were extracted by vigorous shaking with extraction buffer containing 10 % (v/v) glycerol, 0.25 % (w/v) BSA, 0.1 % (v/v) Triton X-100, 50 mM Hepes/KOH, pH 7.5, 10 mM MgCl_2_, 1 mM EDTA, 1 mM EGTA, 1 mM benzamidine, 1 mM ε-aminocapronic acid, 1 mM phenylmethylsulfonyl fluoride, 10 mM leupeptin, and 1 mM DTT [183]. PEPcase was measured spectrophotometrically at 340 nm by coupling the reduction of oxaloacetate by NADH in the presence of malate dehydrogenase (MDH) [184]. The reaction mixture contained enzyme extract, 25 mM Tris-HCl, pH 8, 5 mM MgCl_2_, 4 mM DTT, 5 mM NaHCO_3_, 5 mM glucose-6-phosphate, 5 mM PEP, 0.2 mM NADH and 2 U MDH. RubisCO activity was assayed by coupling RuBP carboxylation to NADH oxidation [185]. The reaction mixture contained enzyme extract, 100 mM bicine/NaOH pH 8, 20 mM MgCl_2_, 25 mM NaHCO_3_, 5 mM phosphocreatine, 3.5 mM ATP, 0.25 mM NADH, 4.8 U G3PDH, 4.8 U creatine phosphofructokinase, 4.8 U G3P kinase and 0.5 mM RuBP. For initial activity, the assay was performed directly after protein extraction. For total activity, leaf extracts were incubated in the assay mix without RuBP for 5 min to fully carbamylate RubisCO [186].

The abundances of PEPcase and RubisCO were estimated by Western Blot [187].

### Total nitrogen, carbon, carbon isotope discrimination and chlorophyll quantification

Powdered dried leaves (3 to 4 mg) were encapsulated in tin capsules and total N, total C and carbon isotope discrimination were evaluated at the UC Davis Stable Isotope Facility. Carbon isotope discrimination was calculated as ∆^13^C according to Farquhar [188]. Chlorophyll was quantified in ethanolic extracts of each leaf segment according to Cross et al. [189].

### Carbohydrates

Soluble sugars were extracted three times with 80 % (v/v) ethanol at 80 °C for 20 min. The supernatants were pooled, dried using a centrifugal vacuum concentrator, resuspended in pure water and filtered. Sugars were separated by high performance anion exchange chromatography with pulsed amperometric detection (HPAEC-PAD, ICS 3000, Thermo Scientific Dionex) on a CarboPac PA-1 4 × 250 mm column set (Thermo Scientific Dionex) using a gradient of eluent A (water) and eluent B (200 mM NaOH), and a flow rate 0.8 mL min^-1^ during 22 min as follows: 0–12 min, 50 % B/50 % A; 12.1–17 min, 100 % B; and 17.1–22 min, 50 % B/50 % A. Myo-inositol, glucose, sucrose and fructose were identified and quantified by comparison with original standards using Chromeleon software (version 6.8, Thermo Scientific Dionex).

### Statistical analysis

All the physiological and biochemical data was subjected analyses of variance (ANOVA) and mean values were compared by the Tukey test (p < 0.05) using the software Origin (OriginLab, USA).

### RNA-Seq

#### RNA extraction, library construction, sequencing and qRT-PCR validation

Total RNA of four independent replicates for each sample was extracted using Trizol (Invitrogen, USA) according to manufacturer’s instructions with an additional sodium acetate/ethanol precipitation step. RNA quality and concentration was assessed by gel electrophoresis, NanoDrop (Thermo Fisher Scientific) and Bioanalyzer (Agilent Technologies). Only RNA samples with a minimum RNA Integrity Number (RIN) of 7 were further processed. Libraries were produced using TruSeq Stranded mRNA Sample Prep Kit (Illumina), which enriches the sample for mRNAs (poly-A containing transcripts) and maintains the information about the strand that was transcribed, according to the manufacturer’s instructions. Clusters were made on c-Bot (Illumina) and paired-end sequencing was carried out on a Hi-Seq 2500 (Illumina) using TruSeq SBS Kit v3 – HS (Illumina). Samples from plants 234, 138 and 163 and DP3 (pool) were sequenced in the LACTAD Facility (University of Campinas, Campinas, Brazil) and samples from an additional biological replicate (plant 235) were sequenced in our institute (CTBE, Campinas, Brazil).

To validate the RNA-seq results, RNA from two additional independent biological replicates were extracted using Trizol (Invitrogen, USA) as mentioned above, and then treated with DNase I Amplification Grade (Invitrogen, USA). cDNA was produced with SuperScript® III (Invitrogen). qRT-PCR was conducted using Sybr Green Master Mix (Applied Biosystems) on an ABI 7500 (Applied Biosystems) real-time PCR system. Primers for 14 selected genes (Table S20 - Additional file 14) were designed using Primer Express 2.0 software and the efficiency of each pair tested using LinRegPCR software [190].

### Transcript assembly and annotation

#### Short read pre-processing and de novo transcript assembly

Short-reads were pre-processed using Trimmomatic v0.32 in order to remove remaining adaptor sequences and to carry out quality trimming, using a sliding window of size 1 bp and a minimum Q value of 20 [191]. In a first iteration, clean reads were *de novo* assembled with Trinity (version r20140413p1) [192, 193], except for reads coming from plant 235, which had not been sequenced at this stage. Possible contaminants were identified using MEGAN5 (http://ab.inf.uni-tuebingen.de/software/megan5/). In a second iteration, reads were mapped against the genomes of representative contaminants, also against mitochondrial (GenBank ACC: NC_008360.1) and plastid (GenBank ACC: NC_005878.2) sequences, and unmapped reads were re-assembled using Trinity. Elimination of contaminating sequences was assessed with MEGAN5 and contigs assigned to Viridiplantae were retained for further analyses, as well as those that did not have any hits against the NCBI nucleotide database, because we can not exclude that these originate from sugarcane.

The quality and completeness of the transcript assembly was evaluated using Transrate [194] by comparing it to the transcripts and proteins in the *Sorghum bicolor* genome [195], and with CEGMA [196], which identifies a set of 248 highly conserved, usually single-copy, eukaryotic genes.

### Transcriptome annotation

Transcript contigs likely originating from sugarcane were annotated with the Trinotate pipeline (http://trinotate.sourceforge.net/), which includes sequence similarity searches against the UniProt and UniRef databases, prediction of signal peptides using SignalP [197], prediction of transmembrane regions using THMM [198] and identification of ribosomal genes with RNAmmer [199]. The KEGG Automatic Annotation Server was used to assign transcripts into KEGG orthologous groups and in house perl scripts were used to retrieve the associated metabolic pathways using KEGG web services [200]. OrthoMCL was employed to identify groups of orthologous genes among sugarcane, *Sorghum bicolor*, *Zea mays*, *Setaria italica*, *Oryza sativa*, and *Arabidopsis thaliana*, using an inflation value of 1.5. The full set of proteins of these species was downloaded from Phytozome [201]. Genes encoding transcription factors and other transcriptional regulators were identified following the approach described in Perez-Rodriguez et al. [66].

### Transcript abundance estimation and differential expression analyses

During the *de novo* assembly by Trinity, the software partitions the input data into many individual de Bruijn graphs, each one representing a single “putative” locus or gene, and then process to split the alternatively spliced forms of that gene [193]. The estimation of transcript abundances was carried out with eXpress [202], summarizing the read counts at the level of gene as defined by Trinity. Differential expression analyses for the leaf +1 developmental stages were carried out with edgeR, controlling for individual/plant variation, i.e., a paired statistical design [203]. Gene counts per sample were normalized using the Trimmed Mean of M-values method – TMM -[204] as implemented in edgeR, in order to account for inter-sample RNA population variation. Only transcripts that achieved 1 count per million, after TMM normalization, in at least three samples were considered as expressed and thus retained for further analysis. Multiple testing was controlled allowing a False Discovery Rate (FDR) of 0.05. Comparisons between segments were performed subtracting distal from basal segments originating the three orthogonal contrasts: Base “zero” vs. Base (B0-B); Middle vs. Base (M-B); Tip vs. Middle (T-M). R code used for differential gene expression analyses is available as Additional file 8.

### Comparison of transcript profiles of orthologous genes between sugarcane and maize along the leaf developmental gradient

We used the expression data (read counts) reported in Wang et al. [48], and processed it in the same way as our sugarcane data (see above). Transcription expression values were reported as FPKM, and in the case of sugarcane we reported the averaged, among replicates, FPKM. We used the list of 30,530 maize and rice orthologous genes from Wang et al. [48] and extracted their orthologous genes in sugarcane for the cases where the orthology relationship was still 1:1 between maize and sugarcane. Finally, the Spearman correlation coefficient between each sugarcane leaf +1 segment and each maize leaf segment was calculated.

### Availability of supporting data

The data sets supporting the results of this article are available in the NCBI’s Short Read Archive (SRA) under the accession numbers: SRR1979656 to SRR1979669 and SRR1974519; the assembled transcriptome is available at NCBI’s Transcriptome Shotgun Assembly (TSA) under accession number: GCZX00000000 and datasets are also available at http://bce.bioetanol.cnpem.br/sugarcanetranscriptome.

## Additional files

Additional file 1:
***It is comprised by 13 Additional figures and methodologies that complement the data presented in the manuscript.*** **Figure S1.** Distribution of leaf length. **Figure S2.** Phenotype of 60 days-old plants. **Figure S3.** Planning for sample collection. **Additional Methodology** of leaf gas exchange and photochemistry evaluation on the B and M segments: on this supplemental topic we described the methodology used to produce Additional Fig. 4 and 5. **Figure S4.** Light response curves for different leaf segments. **Figure S5.** Responses of leaf CO_2_ assimilation to increasing intercellular CO_2_ concentration (C_i_) for different leaf segments. **Figure S6.** Stomata counting in 2 cm segments of sugarcane leaf. **Figure S7.** Venn Diagram representing groups of orthologous genes shared between the five species (*Sorghum bicolor*, *Setaria italica*, *Saccharum* sp. SP80-3280, *Oryza sativa* and *Zea mays*). **Figure S8.** Protein content of sugarcane leaf segments. **Figure S9.** Expression patterns exhibited by the 14 genes identified in all contrasts (Figure 5 on the manuscript). **Figure S10.** Water content. **Figure S11.** Phylogenetic tree of the Trehalose 6-Phosphate Synthase family. **Figure S12.** Expression pattern of cell wall related genes. **Figure S13.** Expression pattern of genes peaking at segment M. **Additional References**. References of the Methodology of Gas exchange and fluorescence evaluation. (DOCX 6742 kb) Additional file 2:
**Fasta file with the assembly.** Transcript assembly as obtained by trinity and submitted to the TSA at NCBI. (BZ2 57956 kb) Additional file 3:
**Assembled transcripts were annotated with Trinotate (** **https://trinotate.github.io/** **).** Transcripts were mapped against the databases UniProt, PFAM, UniRef90. Subcellular location was predicted using SignalP. Transmembrane regions were identify by tmHMM. Ribosomal RNA genes were predicted by RNAMMER. All results were loaded into a SQLite DB as per Trinotate instruction and results were exported into tabular format. For details of the format of this table please check https://trinotate.github.io/. (BZ2 9438 kb) Additional file 4:
**Sugarcane Trinity contigs were mapped to KEGG pathway using the** **single-directional** **best hit approach available in the KEGG Automatic Annotation Service (** **http://www.genome.jp/tools/kaas/** **).** This is a table with the results of such mapping. The table has three columns, 1. The name of the sugarcane transcript/contig, 2. The identifier for the KEGG pathway, and 3. The name of the pathway. (BZ2 76 kb) Additional file 5:
**Transcription associated proteins were identified following the approached presented in Perez-Rodrigues et al 2010.** The table has 5 fields. 1. Gene identifier, as defined in the text, 2. Transcript identifier, 3. Protein identifier, 4. TAP family, 5. This filed has always the value TAP. (BZ2 30 kb) Additional file 6:
**Table with the list of orthologous genes between the five species: Sorghum bicolor (Abbreviation: SBIC), Setaria italica (Abbreviation: SITA), Saccharum sp. SP80–3280 (Abbreviation: SACC), Oryza sativa (Abbreviation: OSAT) and Zea mays (Abbreviation: ZMAY).** Groups of orthologous genes were identified by OrthoMCL with an inflation value of 1.5. (BZ2 1258 kb) Additional file 7:
**This Additional File is an Excel File containing 3 spreadsheets (Additional Tables 1–3) with the results of our differential expression analysis contrasting two leaf segments.** **Table S1.** Differentially expressed genes between the contrast B-B0. **Table S2.** Differentially expressed genes between the contrast M-B. **Table S3.** Differentially expressed genes between the contrast T-M. (XLSX 150 kb) Additional file 8:
**Input data (read count per gene per condition) and R code, to reproduce the differential gene expression analysis for the sugarcane leaf development segments.** (BZ2 15159 kb) Additional file 9:
**Input data and R code to produce the heatmap shown in HeatMapMaize_vs_Sugarcane.** For the comparison, all the orthologous genes used in Wang et al. (2014) that were identified as one to one orthologues between Sugarcane and Maize by OrthoMCL were used. (BZ2 1620 kb) Additional file 10:
**This Additional File is an Excel File containing 6 spreadsheets (Additional Tables 4–9) with the results of our gene ontology analysis for each leaf segment.** **Table S4.** Enriched gene ontology terms for genes over-expressed at B0 identified on the contrast B-B0. **Table S5.** Enriched gene ontology terms for genes over-expressed at B identified on the contrast B-B0. **Table S6.** Enriched gene ontology terms for genes over-expressed at B identified on the contrast M-B. **Table S7.** Enriched gene ontology terms for genes over-expressed at M identified on the contrast M-B. **Table S8.** Enriched gene ontology terms for genes over-expressed at M identified on the contrast T-M. **Table S9.** Enriched gene ontology terms for genes over-expressed at T identified on the contrast T-M. (XLSX 39 kb) Additional File 11:
**This Additional File is an Excel File containing 2 spreadsheets (Additional Tables 10 and 11) showing the identification of genes related to cell wall modification and gene putatively related to suberin biosynthesis.** **Table S10.** Cell Wall related genes identified in each contrast. **Table S11.** Genes putatively involved in suberin biosynthesis identified in each contrast. (XLSX 23 kb) Additional file 12:
**This Additional File is an Excel File containing 6 spreadsheets (Additional Tables 12–17) with the results of our transcriptional factors families enrichment analysis for each leaf segment.** **Table S12.** Transcriptional factors families enriched on B0 considering the contrast B-B0. In red are highlighted the families with significative enrichment. **Table S13.** Transcriptional factors families enriched on B considering the contrast B-B0. In red are highlighted the families with significative enrichment. **Table S14.** Transcriptional factors families enriched on B considering the contrast M-B. **Table S15.** Transcriptional factors families enriched on M considering the contrast M-B. **Table S16.** Transcriptional factors families enriched on M considering the contrast T-M. **Table S17.** Transcriptional factors families enriched on T considering the contrast T-M. In red are highlighted the families with significative enrichment. (XLSX 32 kb) Additional file 13:
**This Additional File is an Excel File containing 2 spreadsheets (Additional Tables 18 and 19) indicating the genes identified as having their peak expression at the middle segment and demonstrating the gene ontology analysis of those genes.** **Table S18.** Genes with expression peaking at the M segment. **Table S19.** Enriched KEGG pathways for genes with expression peaking at the M segment. In grey are highlighted the significant KEGG pathways. (XLSX 92 kb) Additional file 14:
**List of sugarcane genes and primers used for qRT-PCR.** **Table S20.** qRT-PCR Primers. (XLSX 9 kb)

## Abbreviations

A: Photosynthesis
B0: Base “zero” segment
B: Base segment
C_3_: 3-carbon compound
C_4_: 4-carbon compound
DE: Differentially expressed
ETR: Electron transport rate
FPKM: Fragments per kilobase of exon per million fragments mapped
GO: Gene ontology
K: Potassium
*k*: PEPcase: carboxylation efficiency
KEGG: Kyoto encyclopedia of genes and genomes
LogFC: Log Fold Change
M: Middle segment
N: Nitrogen
P: Phosphorus
PEPcase: Phosphoenolpyruvate carboxylase
PEPCK: Phosphoenolpyruvate carboxykinase
PEP: Phosphoenolpyruvate
PNUE: Photosynthetic nitrogen-use efficiency
PSII: Photosystem II
qRT-PCR: Quantitative Real Time PCR
RubisCO: Ribulose-1,5-bisphosphate carboxylase/oxygenase
RuBP: Ribulose-1,5-bisphosphate
T: Tip segment
V_max_: RubisCO capacity
∆^13^C: Carbon isotopic discrimination
