# Supplementary material for: Physiological and transcriptional analyses of developmental stages along sugarcane leaf
Source: BMC Plant Biol. 2015 Dec 29;15:300. doi: 10.1186/s12870-015-0694-z (PMC4696237; doi:10.1186/s12870-015-0694-z)
Supplement: Additional file 1: — It is comprised by 13 Additional figures and methodologies that complement the data presented in the manuscript. Figure S1. Distribution of leaf length. Figure S2. Phenotype of 60 days-old plants. Figure S3. Planning for sample collection. Additional Methodology of leaf gas exchange and photochemistry evaluation on the B and M segments: on this supplemental topic we described the methodology used to produce Additional Fig. 4 and 5. Figure S4. Light response curves for different leaf segments. Figure S5. Responses of leaf CO2 assimilation to increasing intercellular CO2 concentration (Ci) for different leaf segments. Figure S6. Stomata counting in 2 cm segments of sugarcane leaf. Figure S7. Venn Diagram representing groups of orthologous genes shared between the five species (Sorghum bicolor, Setaria italica, Saccharum sp. SP80-3280, Oryza sativa and Zea mays). Figure S8. Protein content of sugarcane leaf segments. Figure S9. Expression patterns exhibited by the 14 genes identified in all contrasts (Figure 5 on the manuscript). Figure S10. Water content. Figure S11. Phylogenetic tree of the Trehalose 6-Phosphate Synthase family. Figure S12. Expression pattern of cell wall related genes. Figure S13. Expression pattern of genes peaking at segment M. Additional References. References of the Methodology of Gas exchange and fluorescence evaluation. (DOCX 6742 kb) [file 12870_2015_694_MOESM1_ESM.docx]

**Additional File 1**

**Figure S1.** Distribution of leaf +1 length of sugarcane. Over 400 plants had their leaf length measured and the frequency distribution was calculated using the square root normal distribution. Only plants with leaf length inside the range of length with the highest frequency (52.3 – 57 cm) were harvested.

**Figure S2.** Phenotype of 60 days old sugarcane plants. Highlight of leaf +1, the first leaf with exposed dewlap.

**Figure S3.** Planning for sample collection. Blue lines indicate the division on three equal thirds. Each third was then divided in half (dashed red lines) and the samples comprised 1 cm of each side (red boxes).We included the first 2 cm of the leaf base – Base “zero”. Segments were named: Base “zero”, base, middle and tip.

**Gas exchange and fluorescence evaluation on B and M segments**

Photosynthetic response curves to light and CO_2_ were performed on B and M segments. A portable photosynthesis system (LI-6400 XT, LiCor, USA) equipped with a fluorometer (LI-6400-40, LiCor) was used for all measurements. Leaf gas exchange and photochemistry was measured varying photosynthetic photon flux density (PPFD) in light response curves (varied from 0 to 2500 µmol m^-2^ s^-1^) or under constant 2000 µmol m^-2^ s^-1^ in CO_2_ response curves. In light response curves, air CO_2_ concentration was maintained at 400 µmol mol^-1^, whereas it varied from 0 to 1700 µmol mol^-1^ in CO_2_ response curves. Leaf temperature was kept at 34±1 °C in all measurements. Simultaneously to the measurement of leaf CO_2_ assimilation (A) and stomatal conductance (g_s_), the PSII operating efficiency (φPSII) was assessed after a saturation pulse (Baker and Oxborough, (2004). Then, the apparent electron transport rate (ETR) was estimated as ETR=φPSII*PARin*0.4*0.85 were φPSII is the PSII operating efficiency; PARin is the photosynthetically active radiation; 0.4 the fraction of absorbed quanta available for photosystem II and 0.85 is the light absorptance by an average green leaf (Rohacek, 2002). Measurements were taken when the total coefficient of variation was lower than 5% and there was temporal stability. Data from light and CO_2_ response curves were used to estimate the PEPcase carboxylation efficiency (*k*) and the maximum RubisCO capacity (V_max_) according to Collatz et al. (1992). *k* and V_max_ were corrected to 25°C (Collatz et al. 1992) and the convexity of light response curve (θ) was estimate through fitting as proposed by Lieth and Reynolds (1987). The convexity of CO_2_ response curve (β) was 0.8 (Marchiori et al., 2014).

Carbon discrimination data was used to calculate leakiness (Φ, the CO_2_ that leaks out from the bundle sheath cells) with equation described by Meinzer et al. (1998). Photosynthetic nitrogen use efficiency (PNUE) was calculated as described by Marchiori et al. (2014), Hirel et al. (2007) and Pons and Westbeek, (2004).

**Figure S4.** Light responses curves for different leaf segments of sugarcane. A: Photosynthesis; B: PSII yield; C: Apperent elctron transport rate; D: Relation between ETR and photosynthesis; Markers with different colors indicate T-test statistical significance (n=4, p≤ 0.05). □ represents the base and ○ represents the middle portions.

**Figure S5.** Responses of leaf CO_2_ assimilation increasing intercellular CO_2_ concentration (C_i_) for different leaf segments if sugarcane. (A) Photosynthetic response - □ represents base and ○ represents the middle. Stomatal conductance (g_S_) at 40 Pa is also presented. B: Maximum RubisCO capacity (V_max_); C: PEPC carboxylation efficiency (*k*). Error bars represents ± SD and letters indicate T-test statistical significance (n=7, p≤ 0.05).

**Figure S6.** Stomata counting in 2 cm segments of sugarcane leaf. Red squares indicate areas of sampling for physiological and transcriptomic analyses (n=5). Stomatal density on each leaf segment was evaluated using a microscope Imager.A2 (Zeiss) with a 50x magnification. A 100W mercury vapor short-arc lamp was employed to emit fluorescent light together with a DAPI filter (365±6 nm). The images were captured and analyzed with the software AxioVision 4.8 (Zeiss).

**
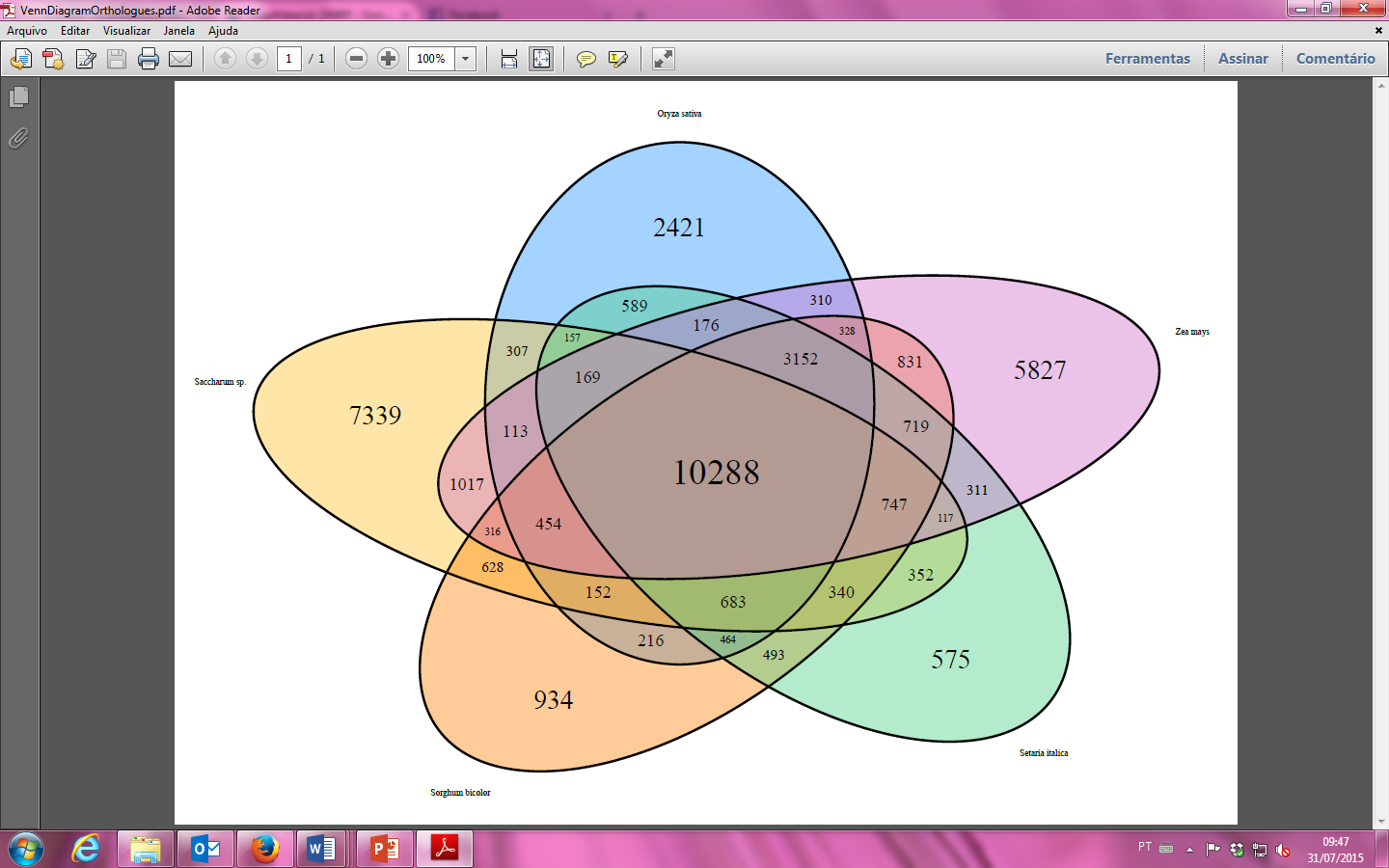
Figure S7.** Venn Diagram representing groups of orthologous genes shared between the five grass species (*Sorghum bicolor*, *Setaria italica*, *Saccharum* sp. SP80-3280, *Oryza sativa* and *Zea mays*). Groups of orthologous genes were identified by OrthoMCL with an inflation value of 1.5

**Figure S8.** Protein content of sugarcane leaf segments. Letters indicate T-test statistical significance (n = 5; p ≤ 0.05).

**Figure S9.** Expression patterns exhibited by the 14 genes identified in all sugarcane leaf segments contrasts (Figure 5 on the manuscript). (A) Pattern 1: Genes that increased its expression from B0 towards the T segments; (B) Pattern 2: Genes that were high at B0 segment, with decreasing expression towards the T; (C) Pattern 3: Genes with lower expression at the M segment; and (D) Pattern 4: Genes with lower expression at B segment. The normalized expression values were obtained by taking the mean of the expression of the gene across the segment, and then take the value for each segment, subtracting the mean and dividing by the standard deviation of the expression of the gene across the segments (Z-value transformation)

**Figure S10.** Water content in sugarcane leaf segments: Base “zero” (B0), Base (B), middle (M) and tip (T). Letters indicate T-test statistical significance between segments (n = 5; p ≤ 0.05).


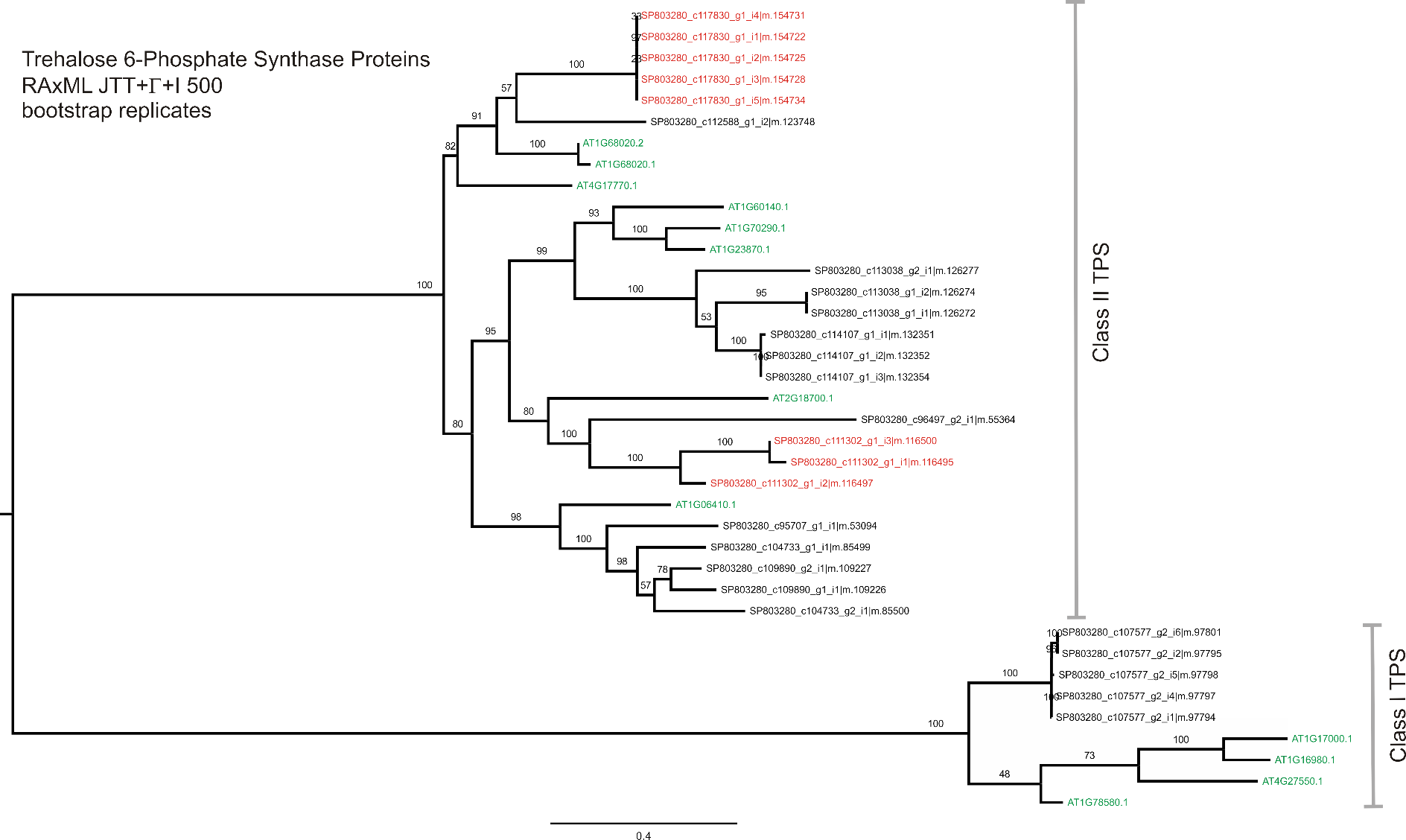


**Figure S11.** Phylogenetic tree of the trehalose 6-phosphate synthase gene family. Multiple protein sequence alignment was carried out with MAFFT v7.245. ProtTest v3.4 was used to determine the appropriate evolutionary model for the family. Phylogenetic inference was carried out in RAxMLv8.2.0 under the evolutionary model JTT+G+I. 500 bootstrap replicates were obtained to assess the confidence of the branches.


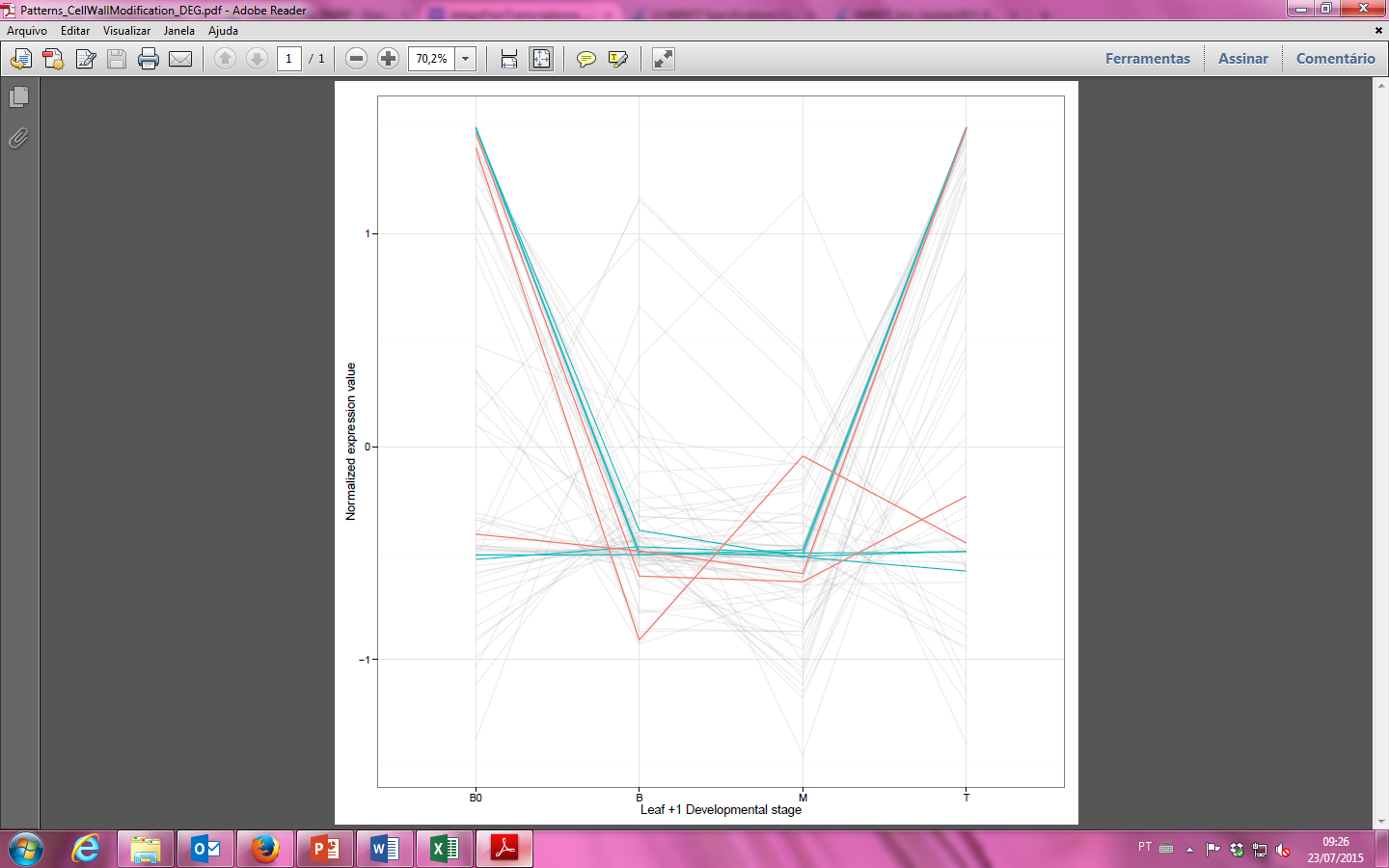


**Figure S12.** Expression pattern of cell wall related genes on sugarcane leaf segments. Blue: COBRA proteins; Red: Expansins. FPKM values were standardized as the number of standard deviations from the mean (Z-value)


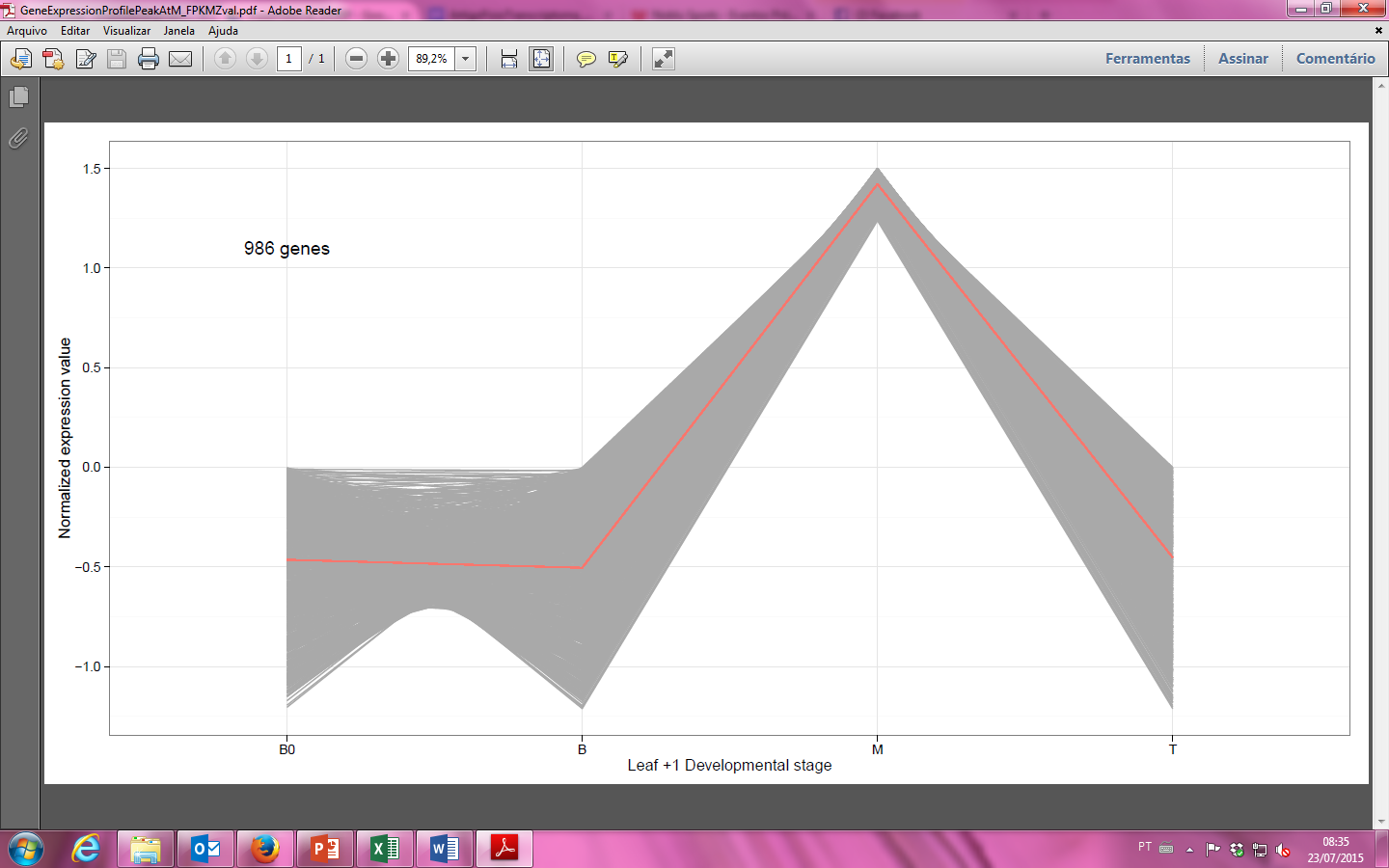


**Figure S13.** Expression pattern of genes peaking at segment M of sugarcane leaves. FPKM values were standardized as the number of standard deviations from the mean (Z-value)

**Additional References**

**Baker, N., and Oxborough, K.** (2004). Chlorophyll fluorescence as a probe of photosynthetic productivity. In Chlorophyll Fluorescence: A Signature of Photosynthesis., P.G. Govindjee, ed (Dordrecht, The Netherlands: Kluwer Academic Publishers), pp. 66-82.

**Collatz, G.J., Ribas-Carbo, M., and Berry, J.A.** (1992). Coupled photosynthesis-stomatal conductance model for leaves of C4 plants. Australian Journal of Plant Physiology **19,** 519-538.

**Hirel, B., Le Gouis, J., Ney, B., and Gallais, A.** (2007). The challenge of improving nitrogen use efficiency in crop plants: towards a more central role for genetic variability and quantitative genetics within integrated approaches. Journal of Experimental Botany **58,** 2369-2387.

**Lieth, J.H., and Reynolds, J.F.** (1987). The nonrectangular hyperbola as a photosynthetic light response model - geometrical interpretation and estimation of the parameter-theta. Photosynthetica **21,** 363-366.

**Marchiori, P., Machado, E., and Ribeiro, V.** (2014). Photosynthetic limitations imposed by self-shading in ﬁeld-grown sugarcane varieties. Field Crops Research 30-37.

**Meinzer, F.C., and Zhu, J.** (1998). Nitrogen stress reduces the efficiency of the C-4 CO2 concentrating system, and therefore quantum yield, in Saccharum (sugarcane) species. Journal of Experimental Botany **49,** 1227-1234.

**Pons, T.L., and Westbeek, M.H.M.** (2004). Analysis of differences in photosynthetic nitrogen-use efficiency between four contrasting species. Physiologia Plantarum **122,** 68-78.

**Rohacek, K.** (2002). Chlorophyll fluorescence parameters: the definitions, photosynthetic meaning, and mutual relationships. Photosynthetica **40,** 13-29.
