# Supplementary material for: Physiological and transcriptional analyses of developmental stages along sugarcane leaf
Source: BMC Plant Biol. 2015 Dec 29;15:300. doi: 10.1186/s12870-015-0694-z (PMC4696237; doi:10.1186/s12870-015-0694-z)
Supplement: Additional file 8: — Input data (read count per gene per condition) and R code, to reproduce the differential gene expression analysis for the sugarcane leaf development segments. (BZ2 15159 kb) [file 12870_2015_694_MOESM8_ESM.bz2 › SugarcaneLeafDevelopment/DiffExpressionAnalysis/DiffExpressionAnalysis.pdf]

Differential Gene Expression analysis - Sugarcane leaf +1 development

Diego M. Riano-Pachon

June, 12, 2015

```
library(edgeR)
```

```
## Loading required package: limma
```

```
rm(list=ls())
setwd("~/SugarcaneLeafDevelopment/DiffExpressionAnalysis")

#Import description of the experiment
targets<-read.table("targets.txt",
                    header=T)
targets
```

```
##    Sample Plant DevStage
## 1    163P   163        T
## 2    235M   235        M
## 3   234Bo   234       Bo
## 4    235P   235        T
## 5    234B   234        B
## 6    138M   138        M
## 7    234P   234        T
## 8    163B   163        B
## 9    138B   138        B
## 10   234M   234        M
## 11  138Bo   138       Bo
## 12   163M   163        M
## 13   138P   138        T
## 14  235Bo   235       Bo
```

```
targets$Plant<-as.factor(targets$Plant)
targets$DevStage<-relevel(targets$DevStage,
                          ref = "Bo")

#Import matrix of RNASeq counts per gene per sample (computed by eXpress)
leaf_dev_counts<-read.table("featurecounts_genelevel.integers.geneLengths.tbl",
                            header=T,
                            row.names='Gene',
                            sep="\t")
dim(leaf_dev_counts)
```

```
## [1] 135481     15
```

```r
head(leaf_dev_counts, n=3L)
```

```
##             X163P X235M X234Bo X235P X234B X138M X234P X163B X138B X234M
## c96089_g1      15     1     10     7    26    19     9     3     8    30
## c100500_g9     43    96     38   229   141   106   130    23    30   140
## c79807_g1      10    31     13   134    39    20    19     6     8    20
##             X138Bo X163M X138P X235Bo Length
## c96089_g1       11    16    16     12    786
## c100500_g9      21    19    77    160   1104
## c79807_g1       22    10    47     84    536
```

```r
leaf_dev_counts_degobj<-DGEList(counts=leaf_dev_counts[,1:14],group=targets$DevStage,
                                genes=data.frame(
                                  GeneID=rownames(leaf_dev_counts),
                                  Length=leaf_dev_counts[,15]))
leaf_dev_counts_degobj$samples
```

```
##        group lib.size norm.factors
## X163P      T 25506670            1
## X235M      M 12427368            1
## X234Bo    Bo 10158356            1
## X235P      T 22383724            1
## X234B      B 32081678            1
## X138M      M 23779625            1
## X234P      T 22713246            1
## X163B      B  5167388            1
## X138B      B 12903763            1
## X234M      M 34250221            1
## X138Bo    Bo 20923858            1
## X163M      M 11956354            1
## X138P      T 38975251            1
## X235Bo    Bo 31743067            1
```

```r
dim(leaf_dev_counts_degobj)
```

```
## [1] 135481     14
```

```r
head(leaf_dev_counts_degobj)
```

```
## An object of class "DGEList"
## $counts
##             X163P X235M X234Bo X235P X234B X138M X234P X163B X138B X234M
## c96089_g1      15     1     10     7    26    19     9     3     8    30
## c100500_g9     43    96     38   229   141   106   130    23    30   140
## c79807_g1      10    31     13   134    39    20    19     6     8    20
## c70915_g1      43    14     24    45    68    43    47    12    32    51
## c119209_g1      0     0      0     0     0     0     2     0     0     0
## c1907_g1        5     5      3    15     7     3     9     1     3     5
##             X138Bo X163M X138P X235Bo
## c96089_g1       11    16    16     12
## c100500_g9      21    19    77    160
```

```
## c79807_g1       22    10    47     84
## c70915_g1       30    13    48     25
## c119209_g1       0     2     0     10
## c1907_g1         6     2    16     22
##
## $samples
##        group lib.size norm.factors
## X163P      T 25506670            1
## X235M      M 12427368            1
## X234Bo    Bo 10158356            1
## X235P      T 22383724            1
## X234B      B 32081678            1
## 9 more rows ...
##
## $genes
##       GeneID Length
## 1  c96089_g1    786
## 2 c100500_g9   1104
## 3  c79807_g1    536
## 4  c70915_g1    918
## 5 c119209_g1    345
## 6   c1907_g1    419
```

```
# Only keep genes that achieve at leat one count per million in at least 3 samples
leaf_dev_counts_degobj_keep <- rowSums(cpm(leaf_dev_counts_degobj)>1) >= 3
leaf_dev_counts_degobj<-leaf_dev_counts_degobj[leaf_dev_counts_degobj_keep,]
dim(leaf_dev_counts_degobj)
```

```
## [1] 40907    14
```

```
# Recompute library sizes after removing "non-expressed genes"
leaf_dev_counts_degobj$samples$lib.size <- colSums(leaf_dev_counts_degobj$counts)
#Compute normalization based due to the differences in the lib. sizes
leaf_dev_counts_degobj<-calcNormFactors(leaf_dev_counts_degobj)
leaf_dev_counts_degobj$samples
```

```
##        group lib.size norm.factors
## X163P      T 25322194    0.7969925
## X235M      M 12173247    1.1088403
## X234Bo    Bo 10019081    1.1221301
## X235P      T 21969611    1.1431309
## X234B      B 31723794    0.9954523
## X138M      M 23592791    0.7787855
## X234P      T 22490059    0.9416446
## X163B      B  5115224    0.9723440
## X138B      B 12750479    0.9291081
## X234M      M 33731002    1.0467761
## X138Bo    Bo 20506751    1.0752220
## X163M      M 11806919    1.0035326
## X138P      T 38557890    0.9355905
## X235Bo    Bo 31032763    1.2657759
```

```
#Create the experimental design table
# The comparison B-Bo is given by coefficient 5, the other comparisons must be made by explicit contras
design<-model.matrix(~Plant+DevStage, data=targets)
leaf_dev_counts_degobj <- estimateGLMCommonDisp(leaf_dev_counts_degobj,design)
leaf_dev_counts_degobj <- estimateGLMTrendedDisp(leaf_dev_counts_degobj,design)
```

```
## Loading required package: splines
```

```
leaf_dev_counts_degobj <- estimateGLMTagwiseDisp(leaf_dev_counts_degobj,design)
barplot(leaf_dev_counts_degobj$samples$lib.size*1e-6,
        names=rownames(leaf_dev_counts_degobj$samples),
        ylab="Library size (millions)",las=2)
```

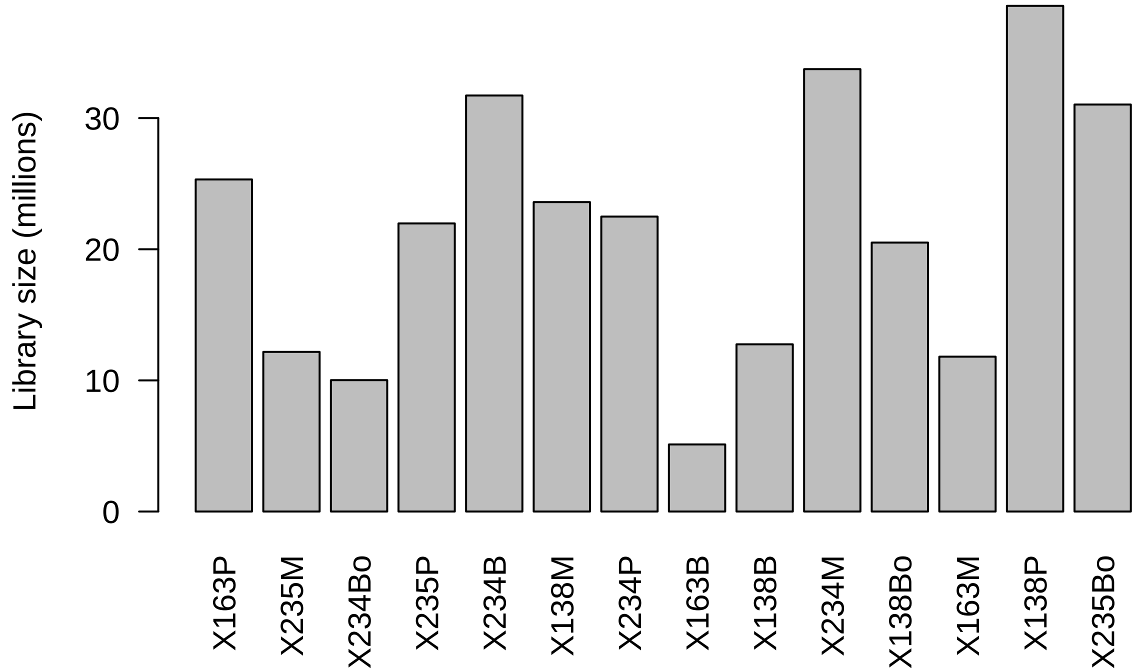


```
plotMDS(leaf_dev_counts_degobj)
```

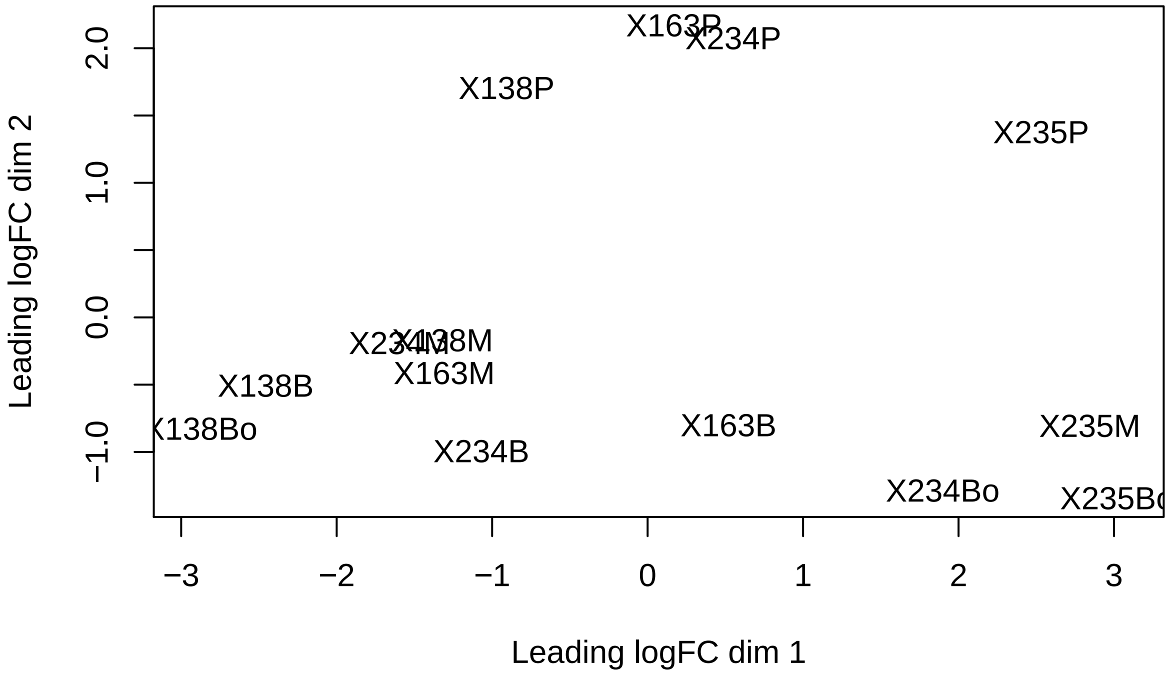


```
plotBCV(leaf_dev_counts_degobj)
```

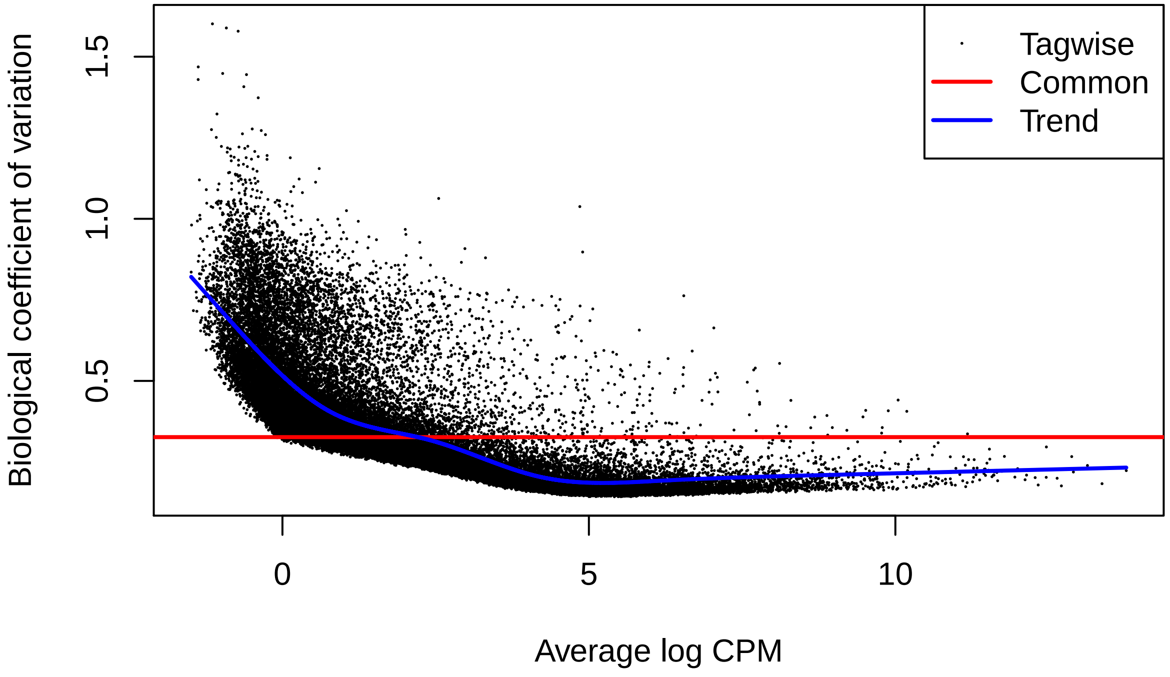


```
plotMeanVar(leaf_dev_counts_degobj, show.raw.vars=TRUE ,
            show.tagwise.vars=TRUE ,
            show.binned.common.disp.vars=FALSE ,
            show.ave.raw.vars=FALSE ,
            NBline = TRUE ,
            nbins = 100 ,
            pch = 16 ,
```

```r
            xlab ="Mean Expression (Log10 Scale)" ,
            ylab = "Variance (Log10 Scale)" ,
            main = "Mean-Variance Plot")
```

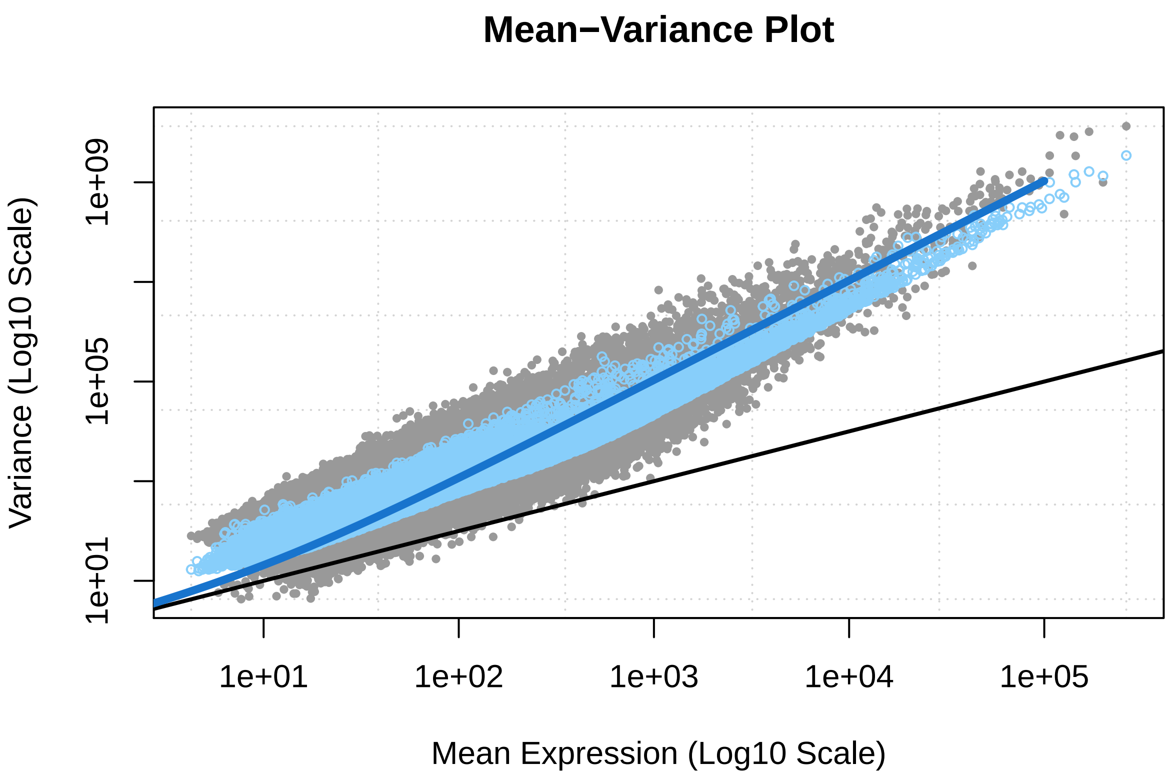


```r
fit_leaf_dev_counts<-glmFit(leaf_dev_counts_degobj, design)
colnames(fit_leaf_dev_counts)
```

```
## [1] "(Intercept)" "Plant163"    "Plant234"    "Plant235"    "DevStageB"
## [6] "DevStageM"   "DevStageT"
```

```r
#Differentially expressed genes in consecutive samples  -  Likelihood ratio test
# B - Bo
lrt_B_Bo<-glmLRT(fit_leaf_dev_counts, coef=5)
# M - B
lrt_M_B<-glmLRT(fit_leaf_dev_counts, contrast=c(0,0,0,0,-1,1,0))
# P - M
lrt_P_M<-glmLRT(fit_leaf_dev_counts, contrast=c(0,0,0,0,0,-1,1))
#Select (index) differentially expressed genes, FDR <=0.05
deB_Bo<-decideTestsDGE(lrt_B_Bo)
deM_B<-decideTestsDGE(lrt_M_B)
deP_M<-decideTestsDGE(lrt_P_M)
#Summary and MA plots
##B - Bo
table(deB_Bo)
```

```
## deB_Bo
##    -1     0     1
##   323 40377   207
```

```
plotSmear(lrt_B_Bo, de.tags=rownames(leaf_dev_counts_degobj)[as.logical(deB_Bo)], ylab = 'Log2FC: B -Bo
abline(h=c(-1, 1), col="blue")
```

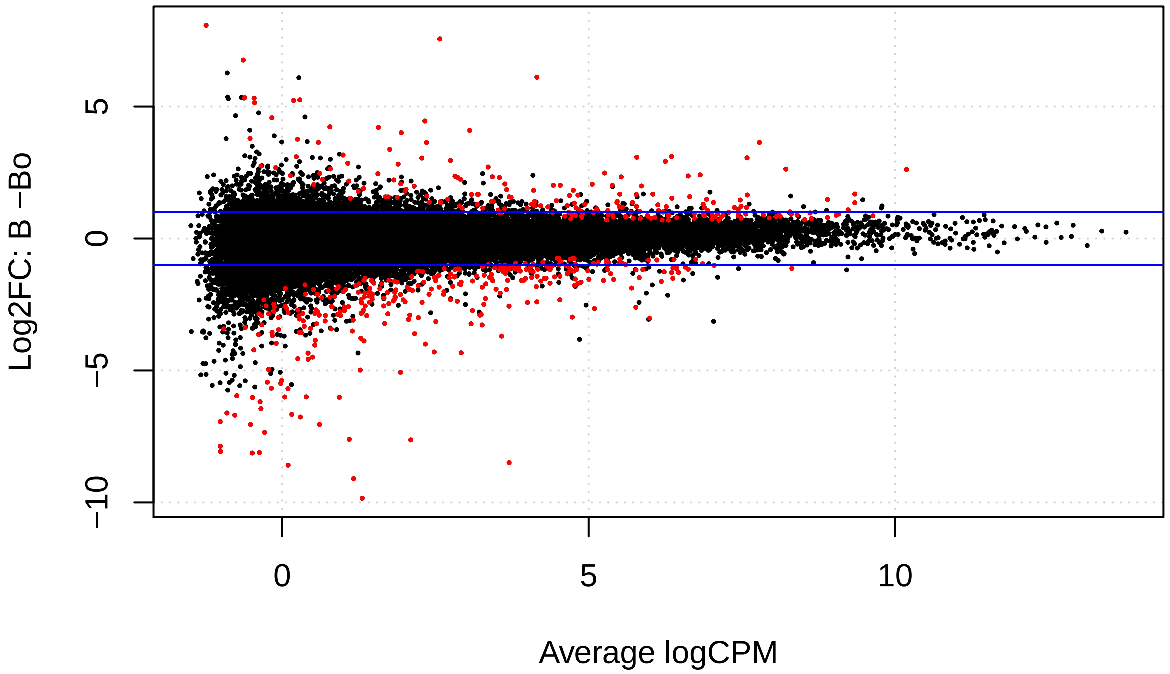


```
##M - B
table(deM_B)
```

```
## deM_B
##    -1     0     1
##    85 40765    57
```

```
plotSmear(lrt_M_B, de.tags=rownames(leaf_dev_counts_degobj)[as.logical(deM_B)], ylab = 'Log2FC: M - B '
abline(h=c(-1, 1), col="blue")
```

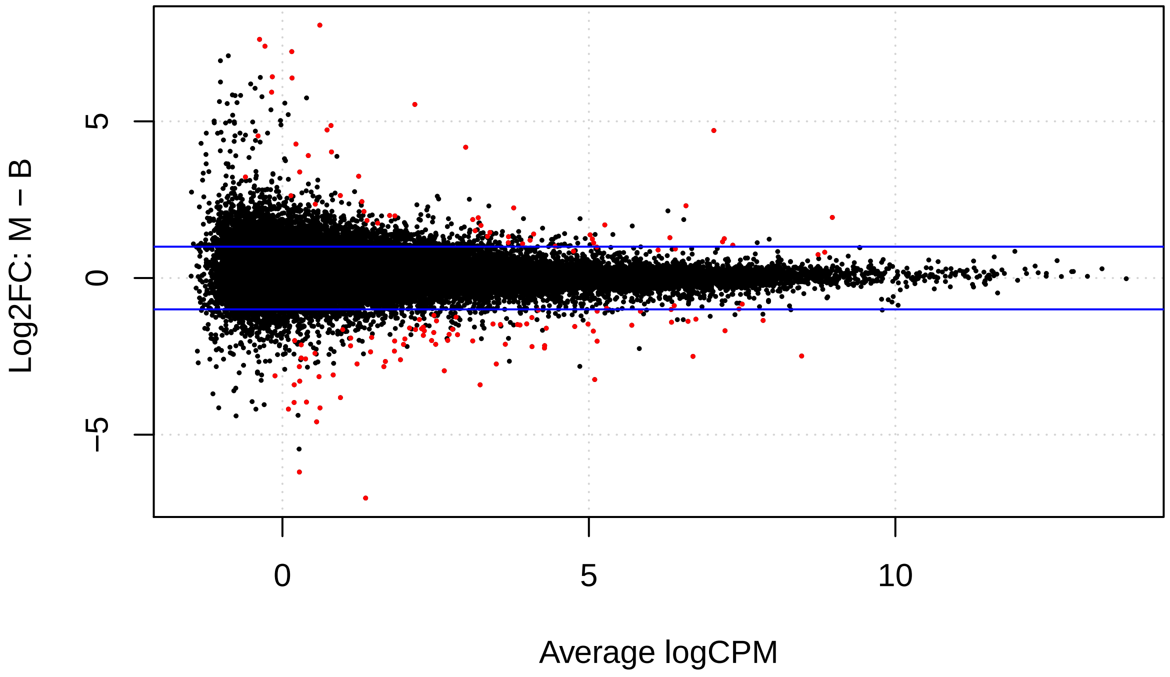


```
##P- M
table(deP_M)
```

```
## deP_M
##    -1     0     1
##   571 39163  1173
```

```
plotSmear(lrt_P_M, de.tags=rownames(leaf_dev_counts_degobj)[as.logical(deP_M)], ylab = 'Log2FC: P - M')
abline(h=c(-1, 1), col="blue")
```

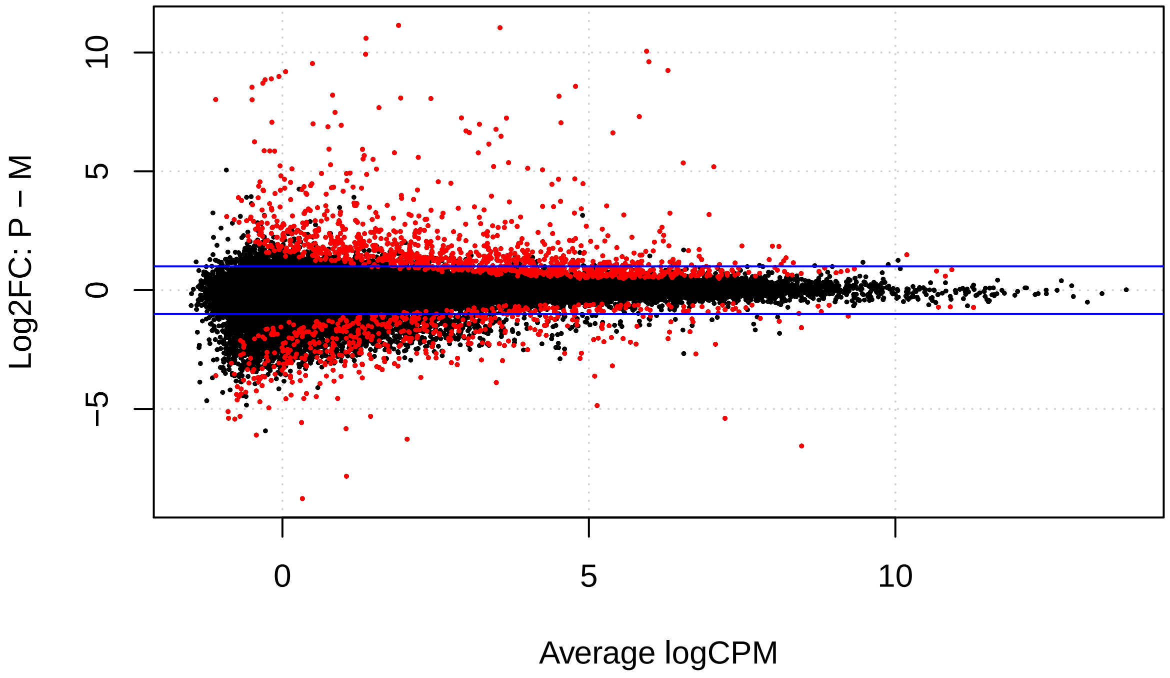

```r
#List of diffexp genes with FDR<=0.05
DEGenesB_Bo<-topTags(lrt_B_Bo,n=as.numeric(table(deB_Bo)[3]+table(deB_Bo)[1]))
DEGenesM_B<-topTags(lrt_M_B,n=as.numeric(table(deM_B)[3]+table(deM_B)[1]))
DEGenesP_M<-topTags(lrt_P_M,n=as.numeric(table(deP_M)[3]+table(deP_M)[1]))

#Exporting tables of DE genes
write.table(DEGenesB_Bo, file="DEGenes_B-Bo.txt")
write.table(DEGenesM_B, file="DEGenes_M-B.txt")
write.table(DEGenesP_M, file="DEGenes_P-M.txt")

#Exporting the expression values as CPM
leaf_dev_counts_degobj_CPM<-cpm(leaf_dev_counts_degobj,log=FALSE)
write.table(leaf_dev_counts_degobj_CPM, file="featurecounts_genelevel.CPM.tbl")

#Exporting the expression values as FPKM
leaf_dev_counts_degobj_FPKM<-rpkm(leaf_dev_counts_degobj,log=FALSE,normalized.lib.sizes=TRUE,prior.coun
write.table(leaf_dev_counts_degobj_FPKM, file="featurecounts_genelevel.FPKM.tbl")

#End
sessionInfo()
```

```
## R version 3.1.2 (2014-10-31)
## Platform: x86_64-unknown-linux-gnu (64-bit)
##
## locale:
##  [1] LC_CTYPE=en_US.UTF-8       LC_NUMERIC=C
##  [3] LC_TIME=en_US.UTF-8        LC_COLLATE=en_US.UTF-8
##  [5] LC_MONETARY=en_US.UTF-8    LC_MESSAGES=en_US.UTF-8
##  [7] LC_PAPER=en_US.UTF-8       LC_NAME=C
##  [9] LC_ADDRESS=C               LC_TELEPHONE=C
## [11] LC_MEASUREMENT=en_US.UTF-8 LC_IDENTIFICATION=C
##
## attached base packages:
## [1] splines   stats     graphics  grDevices utils     datasets  methods
## [8] base
##
## other attached packages:
## [1] edgeR_3.6.8  limma_3.20.9
##
## loaded via a namespace (and not attached):
##  [1] digest_0.6.8    evaluate_0.5.5  formatR_1.0     htmltools_0.2.6
##  [5] knitr_1.9       magrittr_1.5    rmarkdown_0.5.1 stringi_0.4-1
##  [9] stringr_1.0.0   tools_3.1.2     yaml_2.1.13
```
