# Supplementary material for: Physiological and transcriptional analyses of developmental stages along sugarcane leaf
Source: BMC Plant Biol. 2015 Dec 29;15:300. doi: 10.1186/s12870-015-0694-z (PMC4696237; doi:10.1186/s12870-015-0694-z)
Supplement: Additional file 9: — Input data and R code to produce the heatmap shown in HeatMapMaize_vs_Sugarcane. For the comparison, all the orthologous genes used in Wang et al. (2014) that were identified as one to one orthologues between Sugarcane and Maize by OrthoMCL were used. (BZ2 1620 kb) [file 12870_2015_694_MOESM9_ESM.bz2 › SugarcaneLeafDevelopment/ComparisonMaize/ComparingMaizeSugarcane.pdf]

Comparing Maize an Sugarcane leaf development Gene Expression Profiles

Diego M. Riano-Pachon

June, 22nd, 2015

```
library(edgeR)
```

```
## Loading required package: limma
```

```
library(gplots)
```

```
##
## Attaching package: 'gplots'
##
## The following object is masked from 'package:stats':
##
##     lowess
```

```
library(RColorBrewer)
rm(list=c(ls()))
setwd("~/SugarcaneLeafDevelopment/ComparisonMaize")

#import data
#Sugarcane average FPKM
sugarcaneMeanFPKM<-read.table("sugarcane-fpkm.tbl")
head(sugarcaneMeanFPKM)
```

```
##                GeneID        Bo         B         M         T
## 1 SP803280_c100500_g9  2.537936  3.508927  4.177933  4.420754
## 2  SP803280_c79807_g1  2.669485  1.938190  2.236719  3.745884
## 3  SP803280_c70915_g1  1.500273  2.638765  1.611941  2.034961
## 4 SP803280_c100373_g1  5.302502  7.491315  6.930568  7.077695
## 5  SP803280_c98565_g1 37.744771 36.671928 35.679149 36.787622
## 6 SP803280_c104333_g1  1.589904  1.148109  1.602295  1.226343
```

```
#Maize raw data
maize_counts<-read.csv('maize-counts.csv')
maize_lengths<-read.csv('maize-length.csv')

maize_data=merge(maize_counts,maize_lengths,by='Maize.ID')
head(maize_data)
```

```
##           Maize.ID          M1           M2          M3          M4
## 1 AC147602.5_FG004 930.0000000 4711.0000000 7243.000000 7758.000000
## 2 AC148152.3_FG001   0.0000000    1.5000000    0.500000    0.000000
## 3 AC148152.3_FG002   0.5929292    0.5155843    1.183838    1.119625
## 4 AC148152.3_FG005 636.0000000  794.5000000  694.500000  529.500000
```

```
## 5 AC148152.3_FG006   0.0000000    4.2500000   13.000000   18.083333
## 6 AC148152.3_FG007   0.0000000    0.0000000    0.000000    0.000000
##             M5          M6          M7     M8           M9          M10
## 1 1.339400e+04 3124.000000 4008.000000 9772.0 19155.000000 2.525100e+04
## 2 1.500000e+00    1.000000    0.000000    1.0     3.000000 3.000000e+00
## 3 1.909091e-01    0.166667    0.000000    0.0     0.000000 6.818182e-01
## 4 4.395000e+02   70.500000   90.000000  149.0   291.000000 3.010000e+02
## 5 4.833333e+00    1.000000    1.083333    2.5     6.783333 8.250000e+00
## 6 0.000000e+00    0.000000    0.000000    0.0     0.000000 0.000000e+00
##            M11          M12         M13          M14         M15 Length
## 1 1.506900e+04 2.246200e+04 6854.000000 1.167000e+04 5631.000000    483
## 2 2.000000e+00 4.500000e+00    2.000000 0.000000e+00    1.000000   1599
## 3 2.909091e-01 5.909091e-01    0.000000 2.020201e-01    0.000000    390
## 4 2.060000e+02 2.210000e+02   68.000000 1.225000e+02   61.500000   1422
## 5 7.033333e+00 1.073333e+01    5.833332 9.816666e+00    6.983333   1047
## 6 0.000000e+00 0.000000e+00    0.000000 0.000000e+00    0.000000    297
```

```r
rownames(maize_data)<-maize_counts$Maize.ID
maize_degobj<-DGEList(counts=maize_data[,2:16],
                      genes=data.frame(
                        GeneID=maize_data$Maize.ID,
                        Length=maize_data$Length))

maize_degobj$samples
```

```
##     group lib.size norm.factors
## M1      1 11708415            1
## M2      1 16785577            1
## M3      1 18415781            1
## M4      1 15522998            1
## M5      1 16920300            1
## M6      1  2960013            1
## M7      1  3299998            1
## M8      1  7526750            1
## M9      1 14034317            1
## M10     1 20105278            1
## M11     1 13516260            1
## M12     1 20700300            1
## M13     1  6942146            1
## M14     1 12827968            1
## M15     1  6504735            1
```

```r
# Only keep genes that achieve at leat one count per million in at least 3 samples
dim(maize_degobj)
```

```
## [1] 109785     15
```

```r
maize_degobj_keep <- rowSums(cpm(maize_degobj)>1) >= 3
maize_degobj<-maize_degobj[maize_degobj_keep,]
dim(maize_degobj)
```

```
## [1] 27067    15
```

```r
# Recompute library sizes after removing "non-expressed genes"
maize_degobj$samples$lib.size <- colSums(maize_degobj$counts)
#Compute normalization factors based due to the differences in the lib. sizes: TMM normalization
maize_degobj<-calcNormFactors(maize_degobj)
maize_degobj$samples
```

```
##     group lib.size norm.factors
## M1      1 11535834    1.9832868
## M2      1 16672841    1.6577877
## M3      1 18323765    1.4083008
## M4      1 15453348    1.2657214
## M5      1 16854970    1.1268374
## M6      1  2950593    0.9899436
## M7      1  3291079    0.8659348
## M8      1  7505788    0.8382840
## M9      1 13998813    0.7681146
## M10     1 20054055    0.7510260
## M11     1 13482348    0.7377735
## M12     1 20643117    0.7875174
## M13     1  6922044    0.8429308
## M14     1 12787979    0.8572321
## M15     1  6482972    0.8700603
```

```r
#Get FPKM for Maize
maize_degobj_FPKM<-data.frame(rpkm(maize_degobj,
                                   log=FALSE,
                                   normalized.lib.sizes=TRUE,
                                   prior.count=0.25))
maize_degobj_FPKM$Maize.ID=rownames(maize_degobj_FPKM)
head(maize_degobj_FPKM)
```

```
##                          M1         M2         M3         M4          M5
## AC147602.5_FG004 84.159140 352.880334 581.113862 821.186755 1460.070816
## AC148152.3_FG005 19.548930  20.214164  18.926161  19.037313   16.273090
## AC148152.3_FG008  1.632101   5.519172   6.670361  10.488219   14.049991
## AC148167.6_FG001  7.078735  11.497022  11.708164  15.264716   19.573214
## AC149475.2_FG002 79.339123  27.444946  14.300214   5.238405    5.690952
## AC149475.2_FG003 39.109923  25.230597  18.185023  15.575609   10.747563
##                           M6          M7          M8          M9
## AC147602.5_FG004 2214.339403 2911.769766 3215.502629 3688.228549
## AC148152.3_FG005   16.973438   22.208507   16.653258   19.031639
## AC148152.3_FG008   23.696901   24.447561   32.604309   38.051216
## AC148167.6_FG001   26.407500   19.256921   22.769099   17.447710
## AC149475.2_FG002    5.958759    6.539171    8.896638    7.749997
## AC149475.2_FG003   10.369926   13.093075   14.558341   11.063648
##                          M10         M11         M12         M13
## AC147602.5_FG004 3471.157118 3136.524574 2860.657361 2432.039841
## AC148152.3_FG005   14.054316   14.563945    9.559985    8.195643
## AC148152.3_FG008   37.385622   42.255258   41.904803   42.182963
## AC148167.6_FG001   17.540638   17.836218   21.353283   23.308798
## AC149475.2_FG002    7.213925    5.549852    7.461979    8.014857
## AC149475.2_FG003   10.521952    9.929793   10.243110    8.526635
##                          M14         M15         Maize.ID
```

```
## AC147602.5_FG004 2204.059861 2066.878121 AC147602.5_FG004
## AC148152.3_FG005    7.858437    7.667468 AC148152.3_FG005
## AC148152.3_FG008   45.029446   38.751219 AC148152.3_FG008
## AC148167.6_FG001   20.637251   14.981243 AC148167.6_FG001
## AC149475.2_FG002    6.261088    6.420725 AC149475.2_FG002
## AC149475.2_FG003   10.251464    8.223577 AC149475.2_FG003
```

```
#Loading orthologues
orth<-read.table("orthologues_cane-maize_fulllist.tbl")
colnames(orth)<-c('Maize.ID','Sugarcane.ID')
head(orth)
```

```
##        Maize.ID        Sugarcane.ID
## 1 GRMZM2G032409 SP803280_c110522_g1
## 2 GRMZM2G023105 SP803280_c100308_g3
## 3 GRMZM2G093325 SP803280_c105602_g2
## 4 GRMZM2G045892 SP803280_c104301_g1
## 5 GRMZM2G154881 SP803280_c101007_g1
## 6 GRMZM5G891783 SP803280_c110330_g2
```

```
dim(orth)
```

```
## [1] 3789    2
```

```
colnames(sugarcaneMeanFPKM)<-c('Sugarcane.ID','B0','B','M','T')
head(sugarcaneMeanFPKM)
```

```
##          Sugarcane.ID        B0         B         M         T
## 1 SP803280_c100500_g9  2.537936  3.508927  4.177933  4.420754
## 2  SP803280_c79807_g1  2.669485  1.938190  2.236719  3.745884
## 3  SP803280_c70915_g1  1.500273  2.638765  1.611941  2.034961
## 4 SP803280_c100373_g1  5.302502  7.491315  6.930568  7.077695
## 5  SP803280_c98565_g1 37.744771 36.671928 35.679149 36.787622
## 6 SP803280_c104333_g1  1.589904  1.148109  1.602295  1.226343
```

```
OrthologuesFPKM<-merge(orth,sugarcaneMeanFPKM,by='Sugarcane.ID')
OrthologuesFPKM<-merge(OrthologuesFPKM,maize_degobj_FPKM,by='Maize.ID')
dim(OrthologuesFPKM)
```

```
## [1] 2390   21
```

```
head(OrthologuesFPKM)
```

```
##           Maize.ID        Sugarcane.ID         B0          B         M
## 1 AC148152.3_FG008 SP803280_c109324_g2 22.4840317 30.5440434 28.199052
## 2 AC148167.6_FG001  SP803280_c96482_g1 33.9141634 41.3046136 36.723817
## 3 AC149475.2_FG002  SP803280_c93433_g1 22.6873295 20.0396256 14.403565
## 4 AC149818.2_FG006  SP803280_c89040_g1 50.3851165 50.7754665 51.766160
## 5 AC155390.2_FG002 SP803280_c112956_g1  0.6365087  0.5384545  0.507152
## 6 AC155434.2_FG005 SP803280_c110872_g2 12.7609947 13.2532065 15.739021
```

```
##            T         M1         M2         M3         M4         M5
## 1 23.3774338  1.6321008  5.5191723  6.6703611 10.4882191 14.0499907
## 2 33.8841387  7.0787347 11.4970223 11.7081641 15.2647156 19.5732143
## 3 18.2080494 79.3391231 27.4449464 14.3002137  5.2384049  5.6909525
## 4 64.1002086  3.8400594  4.5675117  2.6746278  4.6601836  2.9504974
## 5  0.6038349  0.5031504  0.2535094  0.2133473  0.2814728  0.1844647
## 6 19.7249376  5.8396715  3.6539893  4.1419306  3.0101184  2.2891957
##           M6         M7         M8         M9        M10        M11
## 1 23.6969006 24.4475614 32.6043090 38.0512165 37.3856216 42.2552580
## 2 26.4075005 19.2569213 22.7690989 17.4477104 17.5406385 17.8362183
## 3  5.9587594  6.5391713  8.8966385  7.7499966  7.2139253  5.5498519
## 4  5.0250934  5.9768977  5.4504397  4.6761118  4.4804910  3.4967001
## 5  0.3427006  0.0000000  0.2386373  0.1396396  0.3655443  0.4025371
## 6  0.3101068  0.6356783  0.8637632  1.2635865  2.4056572  2.2765769
##         M12        M13        M14        M15
## 1 41.904803 42.1829626 45.0294463 38.7512194
## 2 21.353283 23.3087980 20.6372510 14.9812428
## 3  7.461979  8.0148565  6.2610882  6.4207249
## 4  4.959754  4.9074626  6.6068850  3.9717971
## 5  0.523381  0.7720061  0.7305066  0.6211250
## 6  1.727258  1.2419229  1.3220582  0.9635154
```

```r
corMaizeSugarcaneOrthologues<-cor(OrthologuesFPKM[, c('B0','B','M','T')],
                                  OrthologuesFPKM[, c(paste('M',1:15,sep=""))],
                                  method='spearman')
heatmap.2(corMaizeSugarcaneOrthologues,
          dendrogram='none',
          scale='none',
          trace='none',
          Rowv=FALSE,
          Colv=FALSE,
          col=brewer.pal(9,"Blues"),
          density.info='none')
```

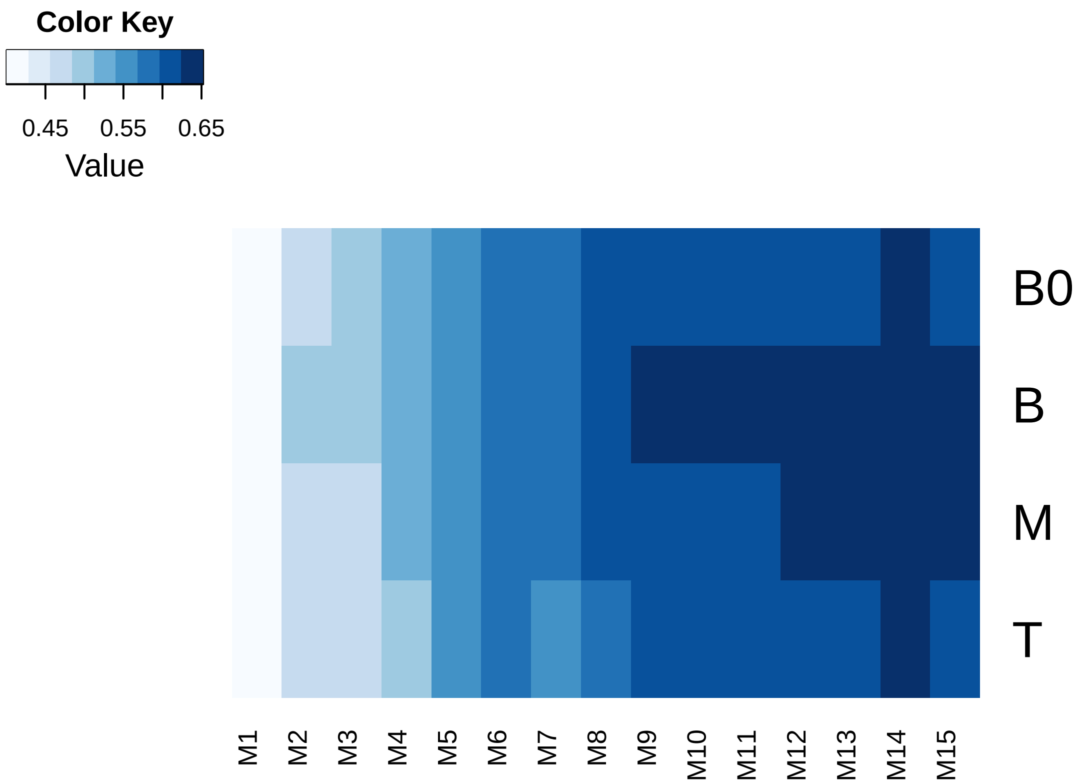


```r
#End
sessionInfo()
```

```
## R version 3.1.2 (2014-10-31)
## Platform: x86_64-unknown-linux-gnu (64-bit)
##
## locale:
##  [1] LC_CTYPE=en_US.UTF-8       LC_NUMERIC=C
##  [3] LC_TIME=en_US.UTF-8        LC_COLLATE=en_US.UTF-8
##  [5] LC_MONETARY=en_US.UTF-8    LC_MESSAGES=en_US.UTF-8
##  [7] LC_PAPER=en_US.UTF-8       LC_NAME=C
##  [9] LC_ADDRESS=C               LC_TELEPHONE=C
## [11] LC_MEASUREMENT=en_US.UTF-8 LC_IDENTIFICATION=C
##
## attached base packages:
## [1] stats     graphics  grDevices utils     datasets  methods   base
##
## other attached packages:
## [1] RColorBrewer_1.1-2 gplots_2.16.0      edgeR_3.6.8
## [4] limma_3.20.9
##
## loaded via a namespace (and not attached):
##  [1] bitops_1.0-6       caTools_1.17.1     digest_0.6.8
##  [4] evaluate_0.5.5     formatR_1.0        gdata_2.13.3
##  [7] gtools_3.4.1       htmltools_0.2.6    KernSmooth_2.23-13
## [10] knitr_1.9          magrittr_1.5       rmarkdown_0.5.1
## [13] stringi_0.4-1      stringr_1.0.0      tools_3.1.2
## [16] yaml_2.1.13
```
